# A Strategy for Enhancement of Power Density in Fe-Based Solvation Difference Flow Battery by Using Solvent that Generates a Large Potential Shift of Ferrocyanide/Ferricyanide

**DOI:** 10.1021/acsomega.5c03793

**Published:** 2025-07-18

**Authors:** Yuki Maeda, Yohei Matsui, Makoto Kawase

**Affiliations:** Energy Chemistry Division, Energy Transformation Research Laboratory, 13329 Central Research Institute of Electric Power Industry, Yokosuka 240-0196, Japan

## Abstract

Effective utilization of thermal energy is attracting attention for the realization of a carbon-neutral society. To convert thermal energy into electrical energy, we recently proposed a solvation difference flow battery (SDFB), a new type of thermally regenerative flow battery, whose electrolyte can be regenerated using thermal energy. The power density of SDFB, however, is relatively low compared with that of the other thermally regenerative flow batteries, and the strategy of the electrolyte design for SDFB to improve the power density has not been clear so far. In this study, we focused on the redox potential of ferrocyanide/ferricyanide in six different solvents (water, ethanol, acetone, 1-butanol, acetonitrile, and dimethyl sulfoxide (DMSO)), where the largest potential difference was recorded between water and DMSO. Density functional theory (DFT) calculations indicated that DMSO had a stronger interaction with ferricyanide than with ferrocyanide, unlike the other solvents, which is the origin of a large potential shift in DMSO. The cell voltage of the water–DMSO SDFB was 1.0 V, which is approximately five times higher than that of the water–acetone SDFB we reported previously. We also investigated the electrochemical behavior of the electrolyte of water–DMSO SDFB and revealed that the electrochemical reaction is quasi-reversible, but the solution and separator resistance were high due to the high viscosity and low conductivity of the water–DMSO solution. As a strategy to decrease the high resistance, we discovered that a porous membrane as the separator was suitable for water–DMSO SDFB, and a maximum power density of 92 W m^–2^ was achieved by decreasing the separator resistance. The maximum power density of the water–DMSO SDFB is more than twice that of the water–acetone SDFB, which we previously reported. In addition, the power density of the water–DMSO SDFB is moderate or high compared to that of the conventional thermally regenerative batteries. This work succeeded in the enhancement of the power density of the SDFB and provides effective insight into the electrolyte design for the improvement of the performance of thermally regenerative flow batteries, including the SDFB.

## Graphical abstract

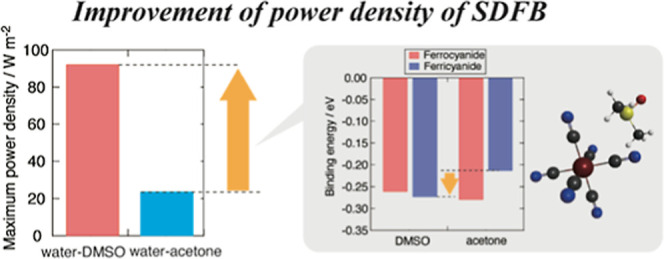

## Introduction

Large quantities of heat sources below 200 °C widely exist in various industries such as steel and chemical production as well as power plants.^1^ Such unused heat sources are expected to be effectively used as a sustainable energy source.^2,3^ Thus, efficient storage of thermal energy and conversion processes of thermal energy into electricity are highly desired to realize a carbon-neutral society. In this situation, several thermo-electrochemical approaches, including thermo-electrochemical cells (TECs),^4^ thermally regenerative electrochemical cycles (TRECs),^5^ and reverse electrodialysis,^6^ have attracted considerable attention as effective approaches for harvesting unused thermal energy. In particular, the thermally regenerative flow battery (TRFB) has attracted attention because TRFB can store thermal energy by reserving the electrolyte, which is charged by thermal energy, and generate electricity by the electrochemical reactions as a flow battery.^7–9^ TRFB usually uses copper (Cu) and ammonia as the redox species and copper ligands, respectively. The Cu-based TRFB has a higher maximum power density (>100 W m^–2^) among other thermo-electrochemical systems.^10,11^ However, an electrochemical system other than the Cu-based system is limited.

Recently, we presented a novel Fe-based TRFB by using not the interaction between redox species and ligands in the conventional TRFBs, but the solvation of the redox species.^12^ We named this TRFB a solvation difference flow battery (SDFB) based on the following concept (Figure 1): The anolyte and catholyte are composed of the same redox species but differ in the solvent composition. The addition of solvent B to solvent A results in a change in the redox potential, owing to fluctuations in the solvation state. The cell voltage of the SDFB is derived from the difference between the redox potentials in the solvents (Figure 1a). By circulation of electrolytes with different solvent compositions between the anolyte and catholyte, a redox reaction can proceed, even though the anolyte and catholyte have the same redox species (Figure 1b). A distinctive feature of SDFB is that the electrolyte can be regenerated through solvent transfer (Figure 1c). For example, if a solvent is transferred via distillation, then the electrolyte can be regenerated using thermal energy. In essence, the SDFB can store thermal energy in electrolytes and convert it into electric energy.

**Figure 1:**
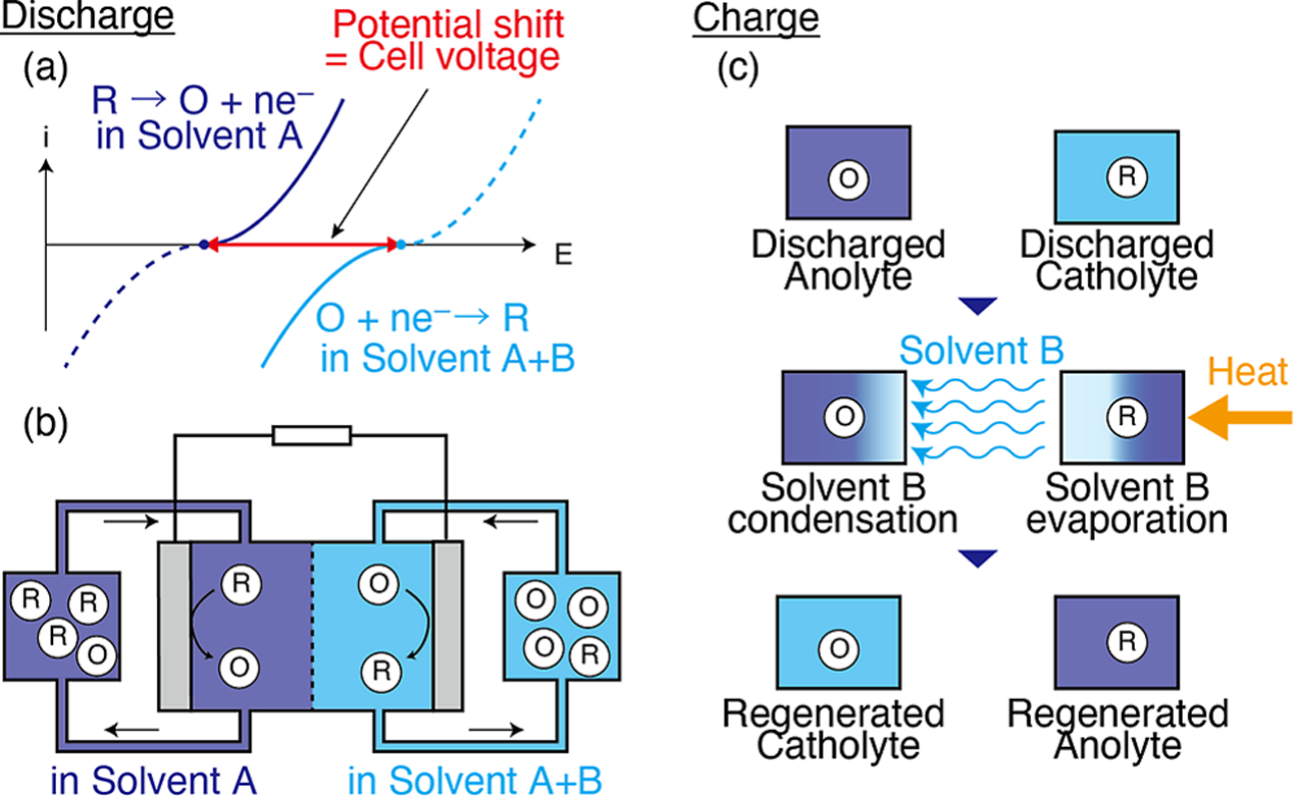
Schematic illustrations of the discharge and charge process of SDFB. O and R denote the oxidant and reductant, respectively. (a) The cell voltage of SDFB derived from the redox potential shift between solvent A and A + B. (b) The schematic illustration of the flow cell based on SDFB. Since the redox potential is positively shifted in solvent A + B, compared with solvent A, solvent A and solvent A + B act as an anolyte and a catholyte of SDFB, respectively. (c) The electrolyte regeneration process of SDFB. The evaporation and condensation of solvent B enable the electrolyte regeneration.

In previous studies, we have proposed Fe-based SDFB using ferrocyanide/ferricyanide as the redox species in water–acetone^13^ and water–2,6-lutidine mixed solvents.^14^ The maximum power densities of the water–acetone and water–2,6-lutidine systems were 40 and 0.15 W m^–2^, respectively. SDFB has a high flexibility of the redox species and solvents compared with the conventional TRFB; however, the power density of SDFB is lower than that of the Cu-based TRFBs. For wider application of an SDFB, a strategy to improve its power density is highly desired.

Our previous research revealed that the cell voltage of an SDFB strongly depends on the combination of solvents used.^12–14^ Thus, the approach for enhancing the power density involves identifying the solvents that generate a large potential shift. In this study, to identify the solvents that generate a high cell voltage for the SDFB, we investigated the redox potential shift of six solvent candidates that are easily available and have low toxicity. The results revealed that the largest potential shift was achieved between water and dimethyl sulfoxide (DMSO). Density functional theory (DFT) calculations showed that DMSO had a strong interaction with ferricyanide and a weak interaction with ferrocyanide, resulting in a significant potential shift in DMSO. In the water–DMSO SDFB, a maximum power density of 92 W m^–2^ was achieved. This value is more than twice that of the water–acetone SDFB.^13^

## Methods

### Electrochemical Measurements

All reagents were purchased from the FUJIFILM Wako Pure Chemical Corporation. The exception was 18-crown 6-ether (18C6), which was purchased from the Tokyo Chemical Industry. Electrochemical measurements were conducted using a three-electrode electrochemical cell and a potentiogalvanostat (Bio-Logic, VSP). A platinum disk electrode with a diameter of 3 mm and a platinum wire were employed as the working electrode (WE) and counter electrode (CE), respectively. A Ag/AgCl electrode in a saturated potassium chloride (KCl) aqueous solution was used as the reference electrode (RE) in mixed solvents with water. For pure solvents, a Ag/Ag^+^ electrode in 0.1 M (M = mol dm^–3^) AgNO_3_ + 0.1 M tetrabutylammonium perchlorate acetonitrile solution was used to suppress the water leakage from RE.

To enhance the solubility of ferrocyanide/ferricyanide in organic solvents, ammonium (NH_4_^+^), tetraethylammonium (TEA^+^), and potassium ion coordinated to 18-crown 6-ether (18C6–K^+^) salts were synthesized using commercially available potassium (K^+^) salts. The NH_4_^+^ and TEA^+^ salts were prepared by using the cation-exchange method with an ion-exchange resin. The details of the synthesis method are described in our previous works.^13,14^ To produce the 18C6–K^+^ salts, the same quantity of 18-crown 6-ether as the K^+^ ion of ferrocyanide/ferricyanide was stirred for several hours in the solvents.

### DFT Calculations

The binding energy (*E*_bind_) between ferrocyanide/ferricyanide and solvents was calculated by DFT using quantum chemical calculation software GAMESS^15^ to investigate the interaction between solvents and ferrocyanide/ferricyanide. *E*_bind_ was determined using eq 1.

1 $$E_{bind}=E_{total}-E_{ion}-E_{solv}$$

where *E*_total_, *E*_ion_, and *E*_solv_ denote the total energy of ferrocyanide/ferricyanide and solvents, the energy of ferrocyanide/ferricyanide, and the energy of solvents, respectively.^16^ The geometry of each structure was optimized and confirmed to be stable via a vibrational analysis. The DFT functional used was the long-range-corrected functional with dispersion correction ωB97X-D.^17^ The basis sets were Lanl2dz for Fe and 6–31+G* for the others. The solvent effects were evaluated by using a polarizable continuum model.

### Power Density Measurement

We investigated the power density of the SDFB using a flow cell. The details of the flow cell have been described in our previous works and Figure S1 in Supporting Information.^13,14^ Carbon paper (SGL group, 39AA, 280 μm thickness) was used for the electrodes, which were heated in an electric furnace at 400 °C in air for 24 h as a pretreatment. The geometric area of the electrodes was 5 cm^2^. The separators of the cell were Nafion membrane (Chemours, NR212, 50 μm thickness) or porous membranes with a porosity of 58% (Daramic, 900 and 175 μm thickness). Nafion membrane was immersed in a 35% tetraethylammonium hydroxide aqueous solution (Tokyo Chemical Industry) for at least 30 min and rinsed with pure water before use. The rinsed separator was sandwiched between the two electrodes. As a flow channel, serpentine electrolyte channels were carved onto the graphitic plates. The anolyte and catholyte were circulated into the flow cell using a tubing pump (As-one, TP-10SA) from the anolyte and catholyte reservoirs.^13^ All experiments were conducted at room temperature. For the measurement of the power density, the polarization curve was obtained in the flow cell.

## Results and Discussion

### Potential Shift in Various Solvents

The potential difference among the solvents is caused by the difference in solvation free energy.^18^ The relationship between the redox potential and solvation energy is described as a Born–Haber cycle (Figure S2).^19,20^ The potential of the redox species in solvent A (*E*_A_) is described in eq 2.

2 $$E_{A}=E_{vac}-\frac{1}{nF}\left(\Delta G_{solv}\left(red,A\right)-\Delta G_{solv}\left(Ox,A\right)\right)$$

where Δ*G*_solv_(red, X) and Δ*G*_solv_(Ox, X) denote the solvation free energies of the reductant and oxidant in solvent X, respectively; *F* and *n* denote the Faraday constant and the number of electrons in the redox reaction (1 for ferrocyanide/ferricyanide), respectively; and *E*_vac_ represents the redox potential in vacuum. From eq 2, the potential difference between solvents A and B (Δ*E* = *E*_A_ – *E*_B_) is expressed by eq 3.

3 $$\Delta E=\frac{-1}{nF}\left\{\Delta G_{solv}\left(red,A\right)-\Delta G_{solv}\left(Ox,A\right)-\Delta G_{solv}\left(red,B\right)+\Delta G_{solv}\left(Ox,B\right)\right\}$$

Equation 3 indicates that the combination of solvents exhibiting a large difference in Δ*G*_solv_(red) – Δ*G*_solv_(Ox) between the solvents generates a high cell voltage for SDFB. In this study, we investigated a combination of solvents that could increase the difference in the free energy of solvation between ferrocyanide and ferricyanide.

In general, solvation free energy is expressed by the Born equation (eq 4)­

4 $$\Delta G_{solv}=-\frac{N_{A}z^{2}e^{2}}{8\pi \varepsilon_{0}r_{0}}\left(1-\frac{1}{\varepsilon_{S}}\right)$$

where *N*_A_, *z*, *e*, ε_0_, *r*_0_, and ε_S_ are the Avogadro constant, charge of the ion, elementary charge, dielectric constant of vacuum, the effective radius of the ion, and dielectric constant of the solvent, respectively. Equation 4 shows that Δ*G*_solv_ is inversely proportional to the dielectric constant of the solvent (ε_S_). In addition to such macroscopic properties of the solvent, Δ*G*_solv_ is affected by the acceptor number and donor number, which are the parameters of electron-accepting and electron-donating properties of the solvent.^18,21^ The acceptor number is defined as the ^31^P NMR chemical shift of triethyl phosphine oxide in the solvent and is used as a parameter for the strength of solvation.^18^ A solvent with a high acceptor number exhibits strong solvation with anions. Thus, the acceptor number and dielectric constant of the solvents are key parameters of the cell voltage. To confirm this assumption, we investigated the relationship between the redox potential and the solvent parameters.

The acceptor numbers, dielectric constants, and boiling points of the candidate solvents are given in Table 1. We chose the solvents that can be transferred by distillation using waste heat at around 200 °C to regenerate the electrolyte. Water, acetonitrile, acetone, 1-butanol, dimethyl sulfoxide (DMSO), and ethanol were selected as candidates because of their low toxicity and ease of availability.

**Table 1:** Physical and Chemical Properties of the Solvents Used in This Work

	acceptor number	dielectric constant	boiling point/°C
water	54.8^a^	78.54	100
acetonitrile	18.9^a^	36.64	81.65
acetone	12.5^a^	21.01	56.05
1-butanol	30.7^b^	17.8	117.7
DMSO	19.3^a^	47	189
ethanol	37.1^a^	24.6	78.5

a Mayer et al., *Monatshefte fur Chemie 106*, 1235 (**1975**).^18^

b Elias et al., *Zeitschrift für Naturforschung B 37*, 684 (**1982**).^28^

To evaluate the potential shift, we measured the redox potentials of mixtures of aqueous solutions with each organic solvent. Mixed solvents were prepared at a mole fraction of the added organic solvent (*x*_s_) of 0.02. K^+^ salts of ferrocyanide/ferricyanide (5 mM) were dissolved in the electrolyte, and the open-circuit potential (OCP) was measured. Cyclic voltammetry (CV) was also performed, and we confirmed that the OCP indicated the redox potential of ferrocyanide/ferricyanide from the CV curve. Figure 2a shows the OCP in water and mixed solvents. The OCP shifted negatively with the addition of organic solvents. The largest potential difference was achieved using water and DMSO. Figure 2b,c shows the relationship between the OCP and acceptor number as well as the dielectric constant of the added solvents. The correlation between the dielectric constant and OCP was found to be negligible. On the other hand, a positive correlation was observed between the acceptor number and OCP. A small acceptor number indicates weak solvation of ferrocyanide/ferricyanide.^18^ This means that when solvent A has a smaller acceptor number than solvent B in eq 3, Δ*G*_solv_(red, A) – Δ*G*_solv_(red, B) > 0 and Δ*G*_solv_(Ox, A) – Δ*G*_solv_(Ox, B) > 0 are established. Moreover, the solvation of ferrocyanide is affected stronger by the solvents than ferricyanide due to the higher charge number of ferrocyanide.^22^ This means that the solvation strongly fluctuates in ferrocyanide. Thus, Δ*G*_solv_(red, A) – Δ*G*_solv_(red, B) > Δ*G*_solv_(Ox, A) – Δ*G*_solv_(Ox, B) are also established. Thus, the redox potential shifts negatively in a solvent with a small acceptor number (Δ*E* < 0).^18^

**Figure 2:**
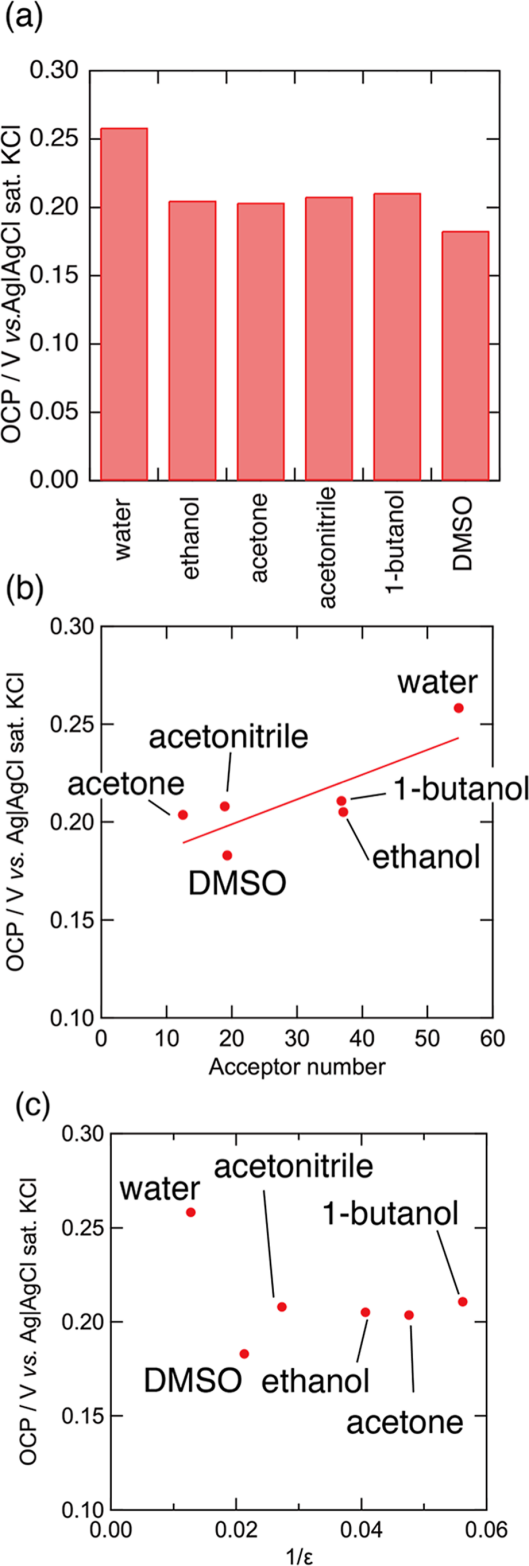
(a) OCPs in water and water–organic solvent mixed solutions with the molar ratio of the organic solvent of 0.02. The OCP dependence of the (b) acceptor number and (c) dielectric constant of the added solvents. *R* ^2^ value of (b) was 0.6476. OCP shifts more negatively by adding the solvent with a smaller acceptor number. On the other hand, no correlation is observed between OCP and the dielectric constant of the added solvents.

In addition, we measured the OCP in pure solvents. The concentrations of both ferrocyanide and ferricyanide were set to 1 mM. Because the solubility of the K^+^ salt in organic solvents is low, we used TEA^+^ salt. The results are shown in Figure 3. Figure 3a shows that the OCP shifted to the most negative potential in DMSO, and a potential difference of approximately 0.9 V was achieved between water and DMSO. These results suggest that a cell voltage of 0.9 V for SDFB should be achieved using a mixed solvent of water and DMSO. This is more than four times larger than the cell voltage of 0.2 V in a water–acetone SDFB reported previously.^13^ Figure 3b,c shows the correlation between the OCP, dielectric constant, and acceptor number. As with the dilute mixed solvent, there was no correlation between the dielectric constant and the OCP. On the other hand, there was also a weak correlation between the OCP and acceptor numbers. Because acetone has the smallest acceptor number among the candidates, we speculated that the potential should shift to the most negative value in acetone. However, DMSO exhibited the largest negative potential shift despite having a higher acceptor number than acetone and acetonitrile.^18^ These results suggest that the unique interactions between ferrocyanide/ferricyanide and DMSO predominantly affect the potential shift.

**Figure 3:**
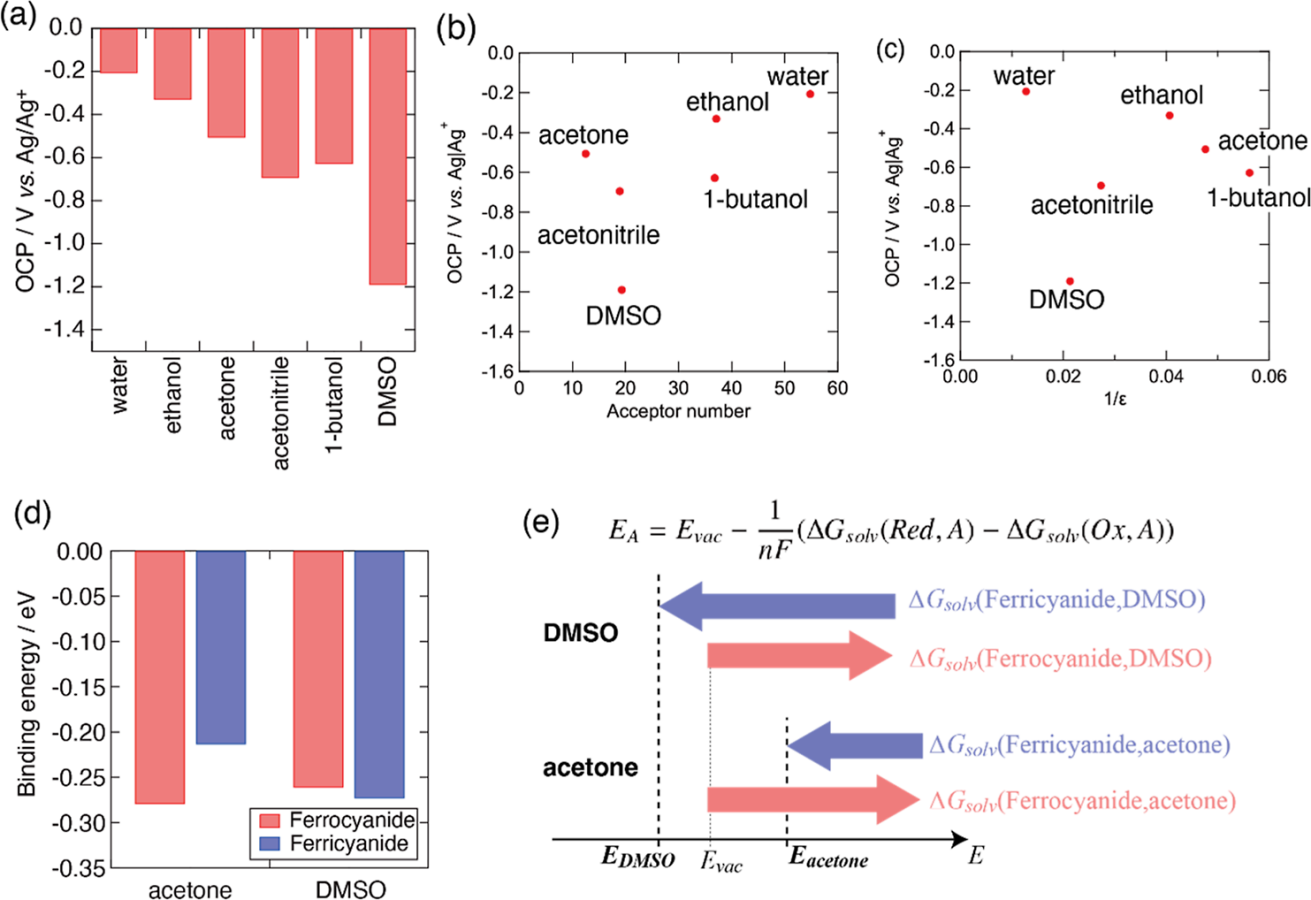
(a) OCPs in water and organic solvent. The OCP dependence of the (b) acceptor number and (c) dielectric constant of the added solvents. No correlation is observed between OCP and the acceptor number, as well as dielectric constant. (d) The binding energy between ferrocyanide/ferricyanide and the solvents calculated by DFT. The magnitude of binding energy between ferrocyanide and acetone is larger than that between ferricyanide and solvent, which is in agreement with the Born equation. On the other hand, in DMSO, ferricyanide has larger binding energy than ferrocyanide, which results in the large negative shift of OCP. (e) The relationship between the Gibbs energy of solvation of ferrocyanide/ferricyanide and the redox potential. The strong interaction between ferricyanide and DMSO results in the negative shift of the redox potential.

To investigate the interaction between ferrocyanide/ferricyanide and the solvents, the binding energy was calculated using DFT. The energy data and optimized geometries are summarized in the Supporting Information. Figure 3d shows the binding energy between ferrocyanide/ferricyanide and the solvents. The magnitude of the binding energy of ferrocyanide was found to be larger than that of ferricyanide in acetone. This trend was also observed in solvents other than DMSO (Figure S3). This is because ferrocyanide has a charge valence higher than that of ferricyanide. However, DFT calculations indicate that only DMSO has a stronger interaction with ferricyanide than with ferrocyanide. This suggests that ferricyanide has a stronger solvation with DMSO than ferrocyanide, unlike those of the other solvents. As a result, the redox potential in DMSO is more negative than that in acetone because of its stronger interaction with ferricyanide than with ferrocyanide (Figure 3e). These results suggest that DFT calculations reveal the unique interaction between ferrocyanide/ferricyanide and solvents, which are not obtained from the acceptor number and can be used to predict the cell voltages of the SDFB.

The origin of the binding energy between ferrocyanide/ferricyanide and the solvents, such as Coulombic interaction, hydrogen bond, van der Waals interaction, and so on, has not been identified by DFT calculation. The optimized geometry from DFT indicates that the closest atoms of the solvent with ferrocyanide/ferricyanide are the H atom of the methyl group in DMSO and acetone. The distance between the N and H atoms is shown in Table S1. The N–H distance of ferrocyanide is closer than that of ferricyanide in the case of acetone. On the other hand, the N–H distance is the same between ferrocyanide and ferricyanide in DMSO. The distance (2.3–2.6 Å) suggests hydrogen bond formation. However, since the polarization of the C–H bond in the methyl group is weak, the effect of the hydrogen bond on the interaction energy is unclear.

In addition, the distance between the Fe atom and C atom of C=O in acetone is almost the same between ferrocyanide and ferricyanide, as shown in Table S2. On the other hand, the distance between the Fe atom and S atom of S=O in DMSO is closer in ferricyanide than in ferrocyanide. This suggests that the interaction between ferricyanide and DMSO is stronger than that between ferrocyanide and DMSO. For further discussion about the interaction between ferrocyanide/ferricyanide and the solvents, the surrounding environment around ferrocyanide/ferricyanide should be considered in more detail by other methods, such as molecular dynamics calculation. To reveal the origin of the interaction, such a detailed analysis of the interaction is required in future work.

Among the candidate solvents, the combination of water and DMSO is expected to significantly improve the cell voltage. Based on these results, we evaluated the performance of the water–DMSO SDFB.

### The Solubility of Ferrocyanide/Ferricyanide in DMSO

We investigated the solubility of ferrocyanide/ferricyanide in a water–DMSO SDFB. Because the solubility in DMSO is insufficient for K^+^ salts, NH_4_^+^, 18C6–K^+^, and TEA^+^ salts were prepared. As for the DMSO-lean solution (*x*_s_ = 0–0.5), 0.1 M aqueous solutions were prepared for each salt, and the OCP was measured by adding DMSO until precipitation occurred. As the DMSO-rich solution (*x*_s_ = 1–0.5), a 0.1 M DMSO solution was prepared, and the OCP was measured while adding water. The change in the OCP for each salt with the mole fraction of DMSO (*x*_s_) is shown in Figure 4. Figure 4 shows that the NH_4_^+^ and 18C6–K^+^ salts precipitated at approximately *x*_s_ = 0.5, and no further OCP change was observed. The solubilities of NH_4_^+^ and 18C6–K^+^ salts are <18.7 mmol kg^–1^ in *x*_s_ = 0.5. The maximum concentration of NH_4_^+^ and 18C6–K^+^ salts in water–DMSO mixed solutions is not enough for the electrolyte of the flow battery. In contrast, the TEA^+^ salt dissolved in the entire solution, and the difference in OCP between *x*_s_ = 0 and 1 was nearly 0.9 V. These results indicate that TEA^+^ salt achieves the highest cell voltage for SDFB due to the high solubility in all composition of DMSO and water.

**Figure 4:**
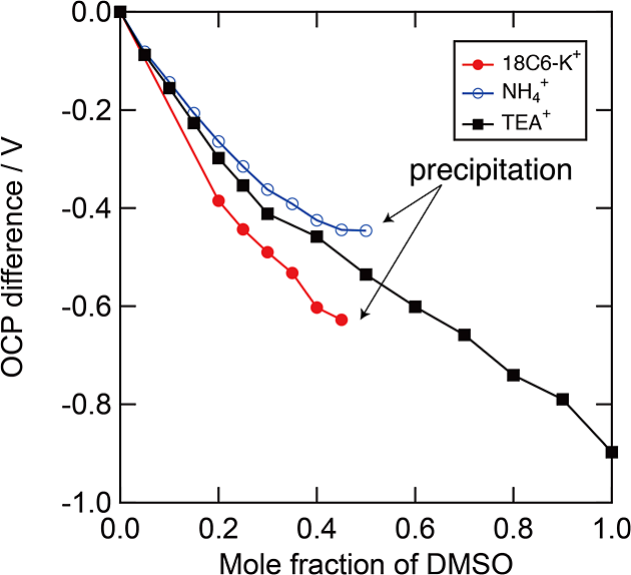
OCP difference in the three types of countercation of ferrocyanide/ferricyanide ions by DMSO addition. 18C6–K^+^ and NH_4_^+^ salts were precipitated in the electrolyte of the mole fraction of DMSO (*x*_s_) = 0.5. On the other hand, TEA^+^ salt was completely dissolved in all electrolyte composition, and a potential shift of 0.9 V is observed between *x*_s_ = 0 and 1.

### Electrochemical Behavior of Ferrocyanide/Ferricyanide in Water–DMSO Mixed Solution

To assess the electrochemical behavior of ferrocyanide/ferricyanide in water–DMSO mixed solution, DMSO-rich (*x*_s_ = 0.9) and DMSO-lean (*x*_s_ = 0.05) solutions were prepared for the TEA^+^ salts, and their electrochemical behaviors were investigated by CV and electrochemical impedance spectroscopy (EIS). The concentration of ferrocyanide/ferricyanide was 0.1 M each, and 0.1 M tetraethylammonium bromide (TEABr) was also added as the supporting electrolyte. The scan rates were 100, 50, 10, and 1 mV s^–1^, and EIS was conducted at frequencies ranging from 1 MHz to 100 mHz. To compare with the water–acetone system, we have previously reported that the same investigations were conducted in the anolyte and catholyte of the water–acetone system where a maximum power density of 40 W m^–2^ was achieved. The anolyte and catholyte composition of the water–acetone system are summarized in Table S3.^13^ In the water–acetone system, NH_4_^+^ salts were used.

Figure 5a shows the CV curves at a scan rate of 50 mV s^–1^. The anodic and cathodic peaks of ferrocyanide/ferricyanide were clearly observed. To assess the reversibility of the redox reactions in water–DMSO and water–acetone systems, the peak separation and the relationship between the peak current and the square root of the scan rate were investigated from the CVs. The results are summarized in Tables S4–S7 and Figures S4 and S5. The linear relationship between the peak current and the square root of the scan rate was observed. Note that the peak separation was larger than the ideal value of the reversible reaction (57 mV) as shown in Tables S4–S7. This is derived from the large solution resistance, as shown in Figure 5b.

**Figure 5:**
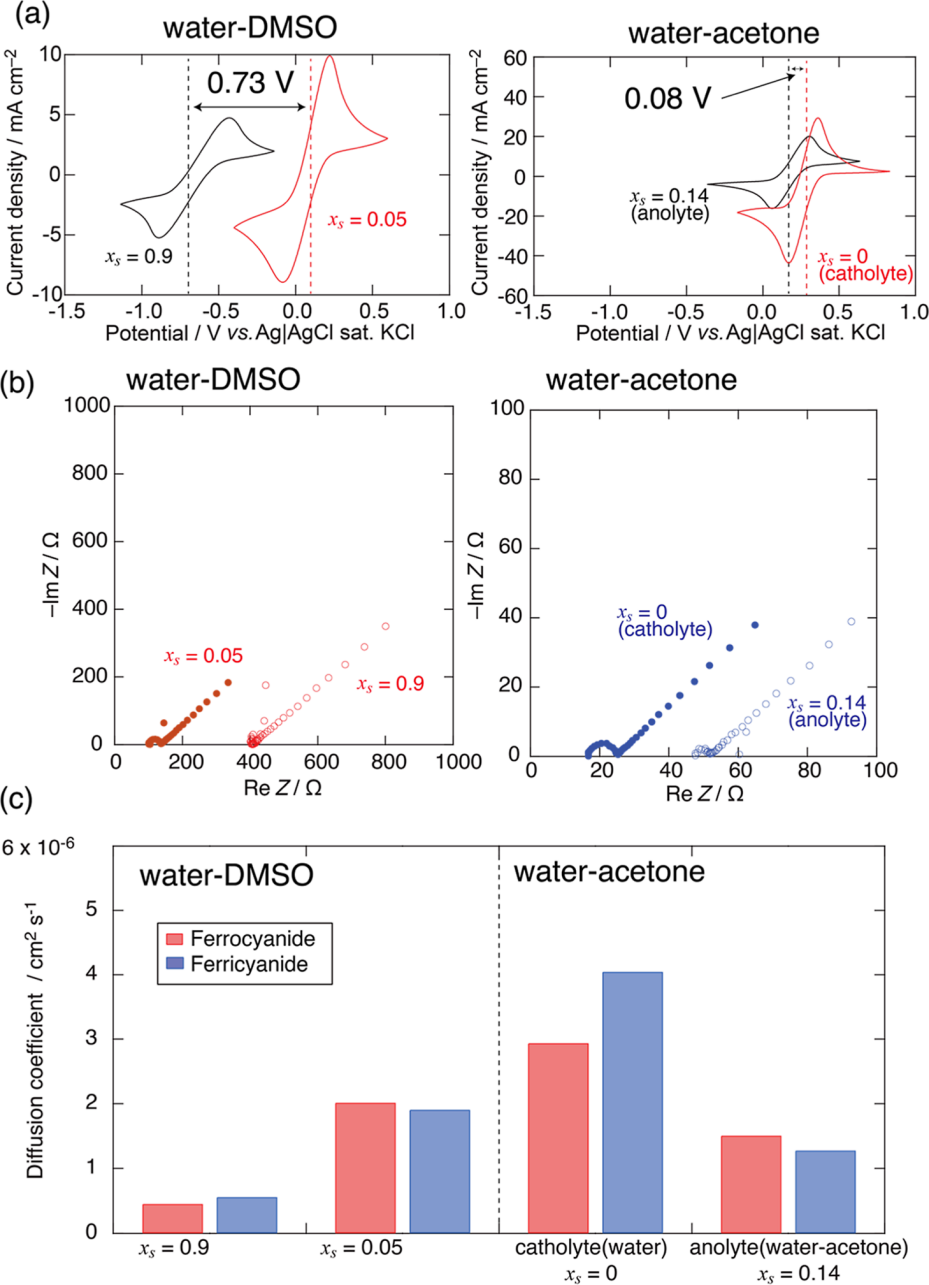
(a) CVs, (b) Nyquist plot, and (c) the diffusion coefficient of ferrocyanide/ferricyanide in water–DMSO mixed solvents of *x*_s_ = 0.05 and 0.9, as well as water–acetone mixed solvents of *x*_s_ = 0 and 0.14. In water–DMSO and water–acetone solutions, the anodic and cathodic peaks are clearly observed, which suggests that the redox reaction of ferrocyanide/ferricyanide is quasi-reversible in water–DMSO and water–acetone solutions. The detailed analysis of CVs is shown in the Supporting Information. The Nyquist plots indicate that the solution resistance of water–DMSO solution is larger than that of water–acetone solution. In addition, the diffusion of ferrocyanide/ferricyanide in water–DMSO solution is slower than that in water–acetone solution.

In addition, to investigate the kinetics of the redox reactions, the standard rate constant (*k* ^0^) was determined from CVs. The detailed methods are described in the Supporting Information.^23^ The results are summarized in Tables S10 and S11. In all electrolytes, the standard rate constants are 10^–4^ cm s^–1^, which means that the electrochemical reactions in water–DMSO mixed solution are quasi-reversible reactions.^23^ Compared with the water–acetone system, the standard rate constant in the water–acetone system is slightly larger than that in the water–DMSO system.

The Nyquist plots are shown in Figure 5b. The fitting methods and results are described in the Supporting Information. The results indicate that the solution resistance of the water–DMSO system is larger than that of the water–acetone system. This is because the molar conductance in water–DMSO mixed solvent extremely decreases as the DMSO concentration increases compared with that in water–acetone mixed solvents.^24,25^

Figure 5c also shows the diffusion coefficient in water–DMSO and water–acetone systems. The results indicate that the diffusion coefficient decreases by adding the organic solvents. Especially, the diffusion coefficient of *x*_s_ = 0.9 in the water–DMSO system was quite low. The viscosity of the mixed solution increased upon the addition of DMSO.^26^ In addition, TEA^+^ also increased viscosity.^27^ Thus, the increase in viscosity due to the addition of DMSO and TEA^+^ inhibited diffusion and reduced the conductivity of the solution. This implies that diffusion and conductivity affect the resistance of the SDFB.

These results indicate that the redox reaction of ferrocyanide/ferricyanide in water–DMSO mixed solvent is quasi-reversible and has a large potential shift, which suggests that the water–DMSO electrolyte achieved SDFB with a large cell voltage. On the other hand, the solution and diffusion resistance are larger than that in water–acetone mixed solvent.

### Power Density of Water–DMSO SDFB

As mentioned above, an improvement in the power density of the water–DMSO SDFB requires a decrease in the diffusion resistance and solution resistance. The effect of diffusion on power density was investigated by examining the effect of the flow rate, which affects the mass transfer of ferrocyanide and ferricyanide to the electrode.

The anolyte and catholyte compositions are listed in Table 2. Since the boiling point of water is lower than that of DMSO, water is the transfer solvent for regeneration of SDFB electrolyte. Thus, weight of DMSO was the same between the anolyte and the catholyte. Since the catholyte was added the transfer solvent (water), the concentration of the redox species and the supporting electrolyte were reduced compared with the anolyte. 1.5 mmol of the redox species was contained in each anolyte and catholyte. The theoretical capacity of the electrolyte was 40.20 mAh. A Nafion membrane was used as the separator. The above discussion suggests that the diffusion rates of ferrocyanide/ferricyanide decreased in the water–DMSO mixed solution. To confirm the effects of ferrocyanide/ferricyanide diffusion on the electrodes, we measured the power densities at flow rates of 10, 22, and 24 mL min^–1^. The minimum and maximum pump flow rates were 10 and 24 mL min^–1^, respectively. Figure 6 indicates that the cell voltage was approximately 1.0 V, which is in good agreement with the OCP difference of the TEA^+^ salt shown in Figure 4. The maximum power densities were 38, 45, and 59 W m^–2^ at 10, 22, and 24 mL min^–1^, respectively. Increasing the flow rate improved the power density owing to the effective diffusion to the electrodes. Although the maximum flow rate of the used pump was 24 mL min^–1^, further improvement of the power density should be achieved by increasing the flow rate.

**Table 2:** Composition of the Electrolytes for the Power Density Measurement^a^

	Anolyte		catholyte	
	weight/g	concentration/mol kg^–1^	weight/g	concentration/mol kg^–1^
water	0		23.41	
DMSO	5.349		5.349	
(TEA)_4_ Fe(CN)_6_	1.099	0.280	0	0
(TEA)_3_ Fe(CN)_6_	0	0	0.9033	0.0521
TEABr	0.5254	0.467	0.5254	0.0869

a For regeneration of the electrolyte by the transfer of water from the catholyte to the anolyte, the amount of DMSO and redox species is the same between the anolyte and the catholyte.

**Figure 6:**
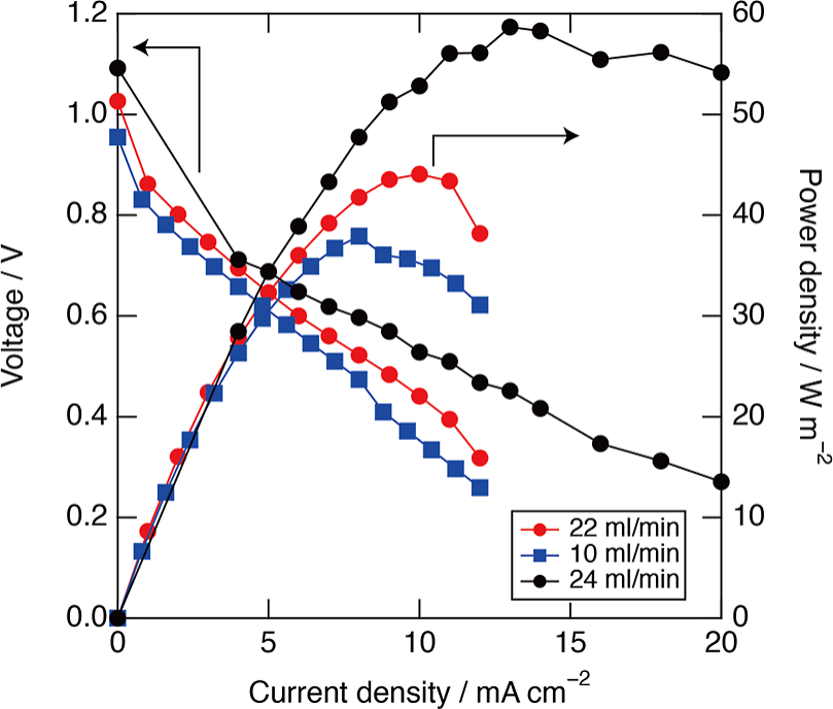
*I*–*V* and power density characteristic with its flow rate at 10, 22, and 24 mL/min. The anolyte and catholyte composition are shown in Table 2. The power density increases as the flow rate increases. The maximum flow rate was 24 mL/min in the pump we used.

In the water–acetone SDFB, the maximum power density was previously reported to be 40 W m^–2^, and the cell voltage was 0.21 V. The cell voltage of the water–DMSO system was about five times higher than that of the water–acetone system, while the maximum power density was about the same. This suggests that a large resistance other than diffusion exists in the water–DMSO SDFB.

We focused on the resistance of the separator and investigated the effects of various separator materials on the power density. In addition to the Nafion membrane, porous membranes with thicknesses of 175 and 900 μm were used as the separators. The flow rate was 24 mL min^–1^. As a comparison, the power density and the resistance in the water–acetone system were also investigated.

Figure 7 shows the values of the real-axis intercept (*R*_s_) of the Nyquist plot and power density for each membrane material, where *R*_s_ indicates the sum of the solution resistance and membrane resistance in the flow cell. The porous membrane (175 μm) in the water–DMSO system has a greatly smaller *R*_s_ than the other membranes, resulting in a maximum power density of 92 W m^–2^. This value is more than twice as high as that of the water–acetone SDFB. In addition, compared to the other thermally regenerative batteries, 92 W m^–2^ in this system is moderate or high power density.^10^ In the case of the Nafion membrane, since the cation-exchange rate of the TEA^+^ ion, which dominates the conductivity in the membrane, is slow, the membrane resistance increases. Although an anion-exchange membrane was also used, the separator resistance was quite high. This implies that Br^–^ did not effectively act as a conduction ion. The reason for this behavior should be investigated in future studies. However, the porous membranes exhibited better ionic conduction because the electrolyte in the porous structure acted as the conduction path. In the porous membranes, the increase in the membrane thickness increased the conduction path, resulting in a high resistance. On the water–acetone system, the smallest *R*_s_ and the largest maximum power density were achieved by using Nafion. This suggests that the suitable separator is different among the solvent combination in SDFB. These results indicate that reducing the membrane resistance is necessary to improve the power density and that a porous membrane with an appropriate thickness is more suitable than an ion-exchange membrane for SDFB.

**Figure 7:**
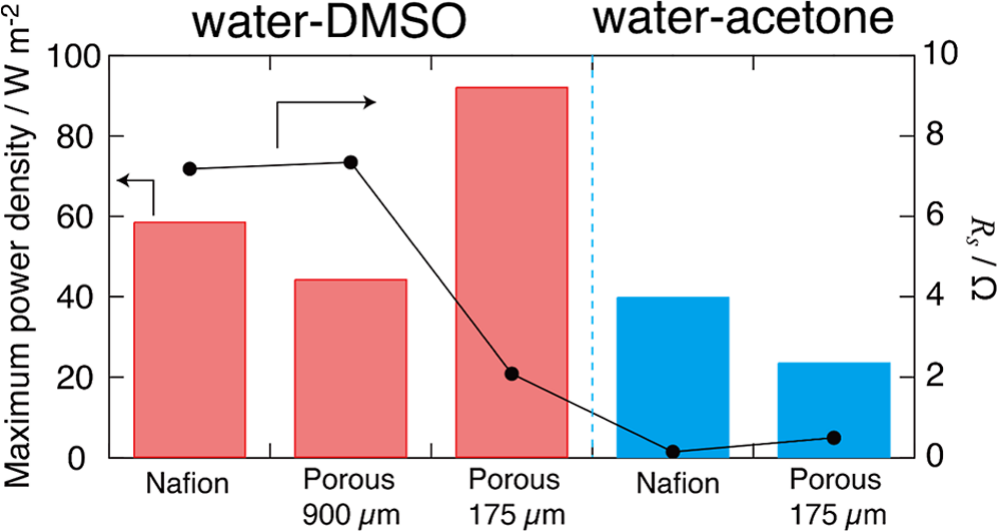
*R*_s_ and maximum power density using Nafion and porous filter (thickness = 900 and 175 μm) as a separator. *R*_s_ is the values of the real-axis intercept of the Nyquist plot in the flow cell, which means that *R*_s_ is the sum of the solution and separator resistance. In water–DMSO solution, a porous separator with a thickness of 175 μm greatly decreases the resistance, which results in a maximum power density of 92 W m^–2^.

The results of the discharge test in water–DMSO SDFB are summarized in the Supporting Information. As shown in Table S14, the potential shift derived from the concentration difference of the redox couple is effectively small compared with the measured open circuit voltage (OCV) in the water–DMSO SDFB. This suggests that the cell voltage is mainly derived from the solvation difference.

Figure S9 demonstrates that the water–DMSO SDFB is capable of thermal regeneration and discharging. Figure S9 also indicates that only 60% of the theoretical capacity is obtained even in the first cycle. This should be due to voltage loss caused by the crossover. Note that the volume of the anolyte (DMSO-rich electrolyte) gradually decreases during discharging in addition to the crossover derived from the concentration gradient. This suggests that the electrolyte crossover also occurs from the anolyte to the catholyte. This crossover is in opposite direction to the crossover driven by the concentration gradient since the concentration of DMSO and ferrocyanide/ferricyanide in the anolyte is larger than that in catholyte. The opposite crossover should be derived from the pressure difference between the anolyte and catholyte flows due to the viscosity difference between the anolyte and catholyte. Unfortunately, we have not resolved the problem by optimizing the flow rate. To improve the discharge properties, the cell design, such as the flow rate and the shape of flow channels, as well as the separator materials, should be modified. The increment of the discharging performance will be our future work.

In addition, since the electrolyte was not completely discharged in the first cycle, the concentration of ferrocyanide/ferricyanide was not completely regenerated even after the solvent separation. Therefore, the normalized capacity after the second cycle is lower than that in the first cycle. On the other hand, the normalized capacity is stable after the second cycle, which suggests that the capacity loss derived from the deterioration of the electrolyte such as deactivation of the redox species is negligible. Thus, the thermal charge/discharge cycle was successfully conducted in the water–DMSO SDFB. The low capacity should be derived from the crossover of the solvents and ferrocyanide/ferricyanide and the difficulty of the complete separation of water from DMSO. To improve the discharge properties, further investigation and optimization of the discharging and regeneration processes should be required in future work.

## Conclusions

To improve the power density of the previously reported SDFB, we investigated a combination of solvents that could increase the cell voltage. The results show that DMSO has the largest potential shift among the candidate solvents, although the acceptor number, which is a key parameter of the solvation energy, suggests that acetone generates the largest potential shift. To investigate the specific behavior of DMSO, the binding energy between the solvent and ferrocyanide/ferricyanide was calculated by using DFT. The results indicate that DMSO has a stronger interaction with ferricyanide than ferrocyanide, unlike the other solvents. A large potential shift in DMSO is derived from the unique interaction behavior with ferrocyanide/ferricyanide. The results suggest that DFT calculations can be used to predict the cell voltage of the SDFB and provide new insights into solvation, which cannot be obtained by the acceptor number.

In a DMSO–water SDFB, the cell voltage exceeded 1.0 V, which is five times larger than a water–acetone SDFB. Despite the high cell voltage, the power density was not high owing to the high cell resistance derived from the separator. By optimization of the separator materials, a maximum power density of 92 W m^–2^ was achieved. This value is more than twice that previously reported for a water–acetone SDFB. In addition, the power density of the water–DMSO SDFB is moderate or high compared with that of the conventional thermally regenerative batteries.

This work suggests that the interaction of redox species and solvents has a significant effect on the potential shift of ferrocyanide/ferricyanide. This provides effective insight into the electrolyte design for improvement of the performance of thermally regenerative flow batteries including SDFB. Further improvement of the performance is highly required to utilize SDFB as an energy harvesting system from waste heat. This work revealed that the main barrier to improving the performance of SDFB is derived from the high cell resistance of the water–organic mixed solvents and the crossover. In future work, we will investigate the suitable cell design for SDFB, other combinations of the redox species and solvents that generate high power density, and effective solvent separation to improve the SDFB performance.

## Supplementary Material



## Notes

Additional experimental details, DFT calculation data, detailed analysis of CVs and EISs, and thermal charge/discharge properties of water–DMSO SDFB (PDF)
