# Supplementary material for: A Strategy for Enhancement of Power Density in Fe-Based Solvation Difference Flow Battery by Using Solvent that Generates a Large Potential Shift of Ferrocyanide/Ferricyanide
Source: ACS Omega. 2025 Jul 18;10(29):32070–9. doi: 10.1021/acsomega.5c03793 (PMC12311857; doi:10.1021/acsomega.5c03793)
Supplement: Supplementary file 1 [file ao5c03793_si_001.pdf]

Supporting information

Strategy for Enhancement of Power Density in Fe-based Solvation Difference Flow Battery by Using Solvent that Generates a Large Potential Shift of Ferrocyanide/Ferricyanide

Yuki Maeda, Yohei Matsui, Makoto Kawase

Energy Chemistry Division, Energy Transformation Research Laboratory, Central Research Institute of Electric Power Industry, Yokosuka, 240-0196, Japan

Experimental

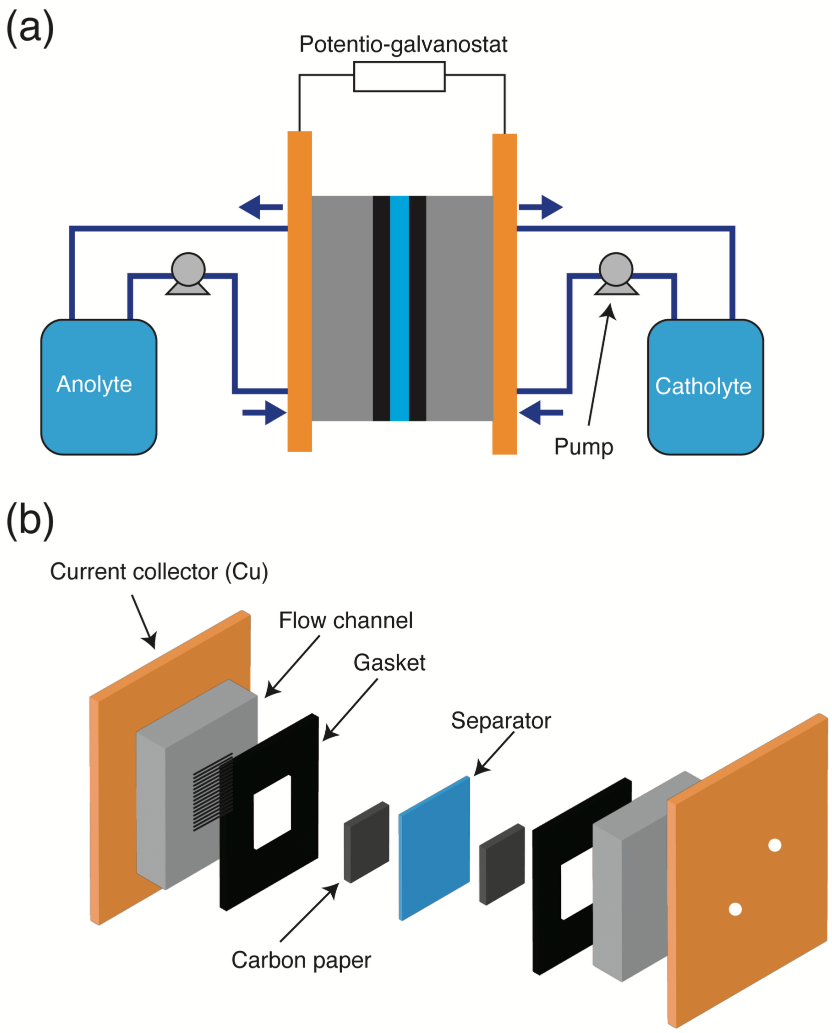

Figure S1. Schematic illustrations of (a) the used flow cell and (b) the flow cell composition. Anolyte and catholyte were circulated into the flow cell using a tubing pump from the reservoirs. The current collector (gold-coated Cu plate), graphite flow channel, gasket (Viton®, 0.3 mm-thick), carbon paper electrode (5 cm^2), and separator were sandwiched.

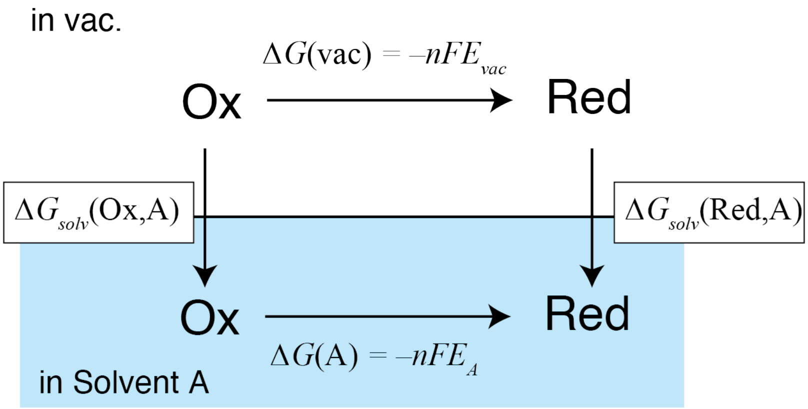

Figure S2. The Born-Haber cycle of the redox reaction.

DFT calculations

Energy data

		Energy / Hartree			Binding energy / Hartree
solute	solvent	solute-solvent	solute	solvent	
$Fe(CN)_6^{4-}$	acetone	-874.024	-680.906	-193.107	-0.01028
$Fe(CN)_6^{3-}$		-873.899	-680.783		-0.00785
$Fe(CN)_6^{4-}$	DMSO	-1234.08	-680.906	-553.139	-0.26141
$Fe(CN)_6^{3-}$		-1233.95	-680.783		-0.27319
$Fe(CN)_6^{4-}$	ethanol	-835.925	-680.906	-154.996	-0.40298
$Fe(CN)_6^{3-}$		-835.797	-680.783		-0.36278
$Fe(CN)_6^{4-}$	acetonitrile	-813.655	-680.906	-132.719	-0.25408
$Fe(CN)_6^{3-}$		-813.521	-680.783		-0.18709
$Fe(CN)_6^{4-}$	1-butanol	-914.519	-680.906	-233.605	-0.4768
$Fe(CN)_6^{3-}$		-914.397	-680.783		-0.35485
$Fe(CN)_6^{4-}$	water	-757.358	-680.906	-76.3968	-0.5336
$Fe(CN)_6^{3-}$		-757.215	-680.783		-0.4122

XYZ coordinates of optimized geometries

$Fe(CN)_6^{4-}$ acetone

ATOM	CHARGE	X	Y	Z
FE	26	-0.7359941	0.43059333	0.33360596
O	8	-6.0566849	-0.1342149	-3.3499503
N	7	-3.0295581	2.50227623	-0.2686007
N	7	-2.6373889	-2.0313244	-0.1569506
N	7	-1.6790149	0.50003584	3.31604365
N	7	1.67731777	-1.5056978	0.99323762
N	7	1.23421787	2.82391425	0.84751194
N	7	0.22483593	0.3611495	-2.6478079
C	6	-2.1775057	1.71227134	-0.0783336
C	6	0.7526275	-0.8131982	0.76415636
C	6	-1.9315678	-1.1021036	0.00198407
C	6	0.48244511	1.93290056	0.68027608
C	6	-1.3068595	0.44381741	2.19993789
C	6	-0.1639232	0.41559469	-1.53772
C	6	-5.3883503	-0.000841	-1.0591266
C	6	-5.137736	-0.1191537	-2.5359447
C	6	-3.6990464	-0.2198379	-2.9575646
H	1	-6.457413	-0.0458656	-0.8383736
H	1	-4.9566192	0.94380384	-0.703473
H	1	-4.8378682	-0.7871743	-0.5308123
H	1	-3.1033302	0.56466179	-2.4778763
H	1	-3.6087678	-0.1774994	-4.0453381
H	1	-3.2845612	-1.1584967	-2.572925

$Fe(CN)_6^{3-}$ acetone

ATOM	CHARGE	X	Y	Z
FE	26	-0.4348292	1.23312367	-0.509078
C	6	-1.1943805	2.86857272	0.26516751
C	6	0.33855107	-0.393871	-1.2817805
C	6	-2.1776693	0.8915732	-1.3375967
C	6	1.29278106	1.59486588	0.34755794
C	6	-0.9780832	0.2653133	1.11158871
C	6	0.05121716	2.15759743	-2.1642124
N	7	-1.6246979	3.85209702	0.72651363
N	7	-3.2211865	0.71363723	-1.8336928
N	7	-1.3153219	-0.3060356	2.07366631
N	7	0.76974748	-1.3770979	-1.7444528
N	7	2.30161911	1.84759204	0.88099738
N	7	0.33097069	2.70614093	-3.1573329
C	6	1.63718504	5.1701543	-0.8322794

C	6	3.00307061	5.11450584	-1.4624999
C	6	3.46349059	3.76685416	-1.9494114
H	1	0.88083916	4.90856988	-1.5812666
H	1	1.43611091	6.16412276	-0.4269264
H	1	1.55004487	4.40878065	-0.0499513
H	1	3.48093578	3.06270795	-1.1091888
H	1	4.45073881	3.83608072	-2.4113163
H	1	2.72932577	3.36745439	-2.6582898
O	8	3.70217511	6.11483415	-1.5779194

$Fe(CN)_6^{4-}$ DMSO

**ATOM**	CHARGE	X	Y	Z
FE	26	-2.6304197	2.42036643	0.29984138
S	16	1.59835295	6.19185476	0.12449769
O	8	3.0434143	6.34742849	-0.345834
N	7	-2.7049245	5.37782402	-0.717358
N	7	-5.4871545	2.74415396	1.56407595
N	7	-4.0619405	1.3660516	-2.3143327
N	7	0.18671431	1.88056258	-0.9841419
N	7	-1.3480991	3.51735071	2.96546466
N	7	-2.5769362	-0.5441929	1.30330062
C	6	-2.6771446	4.25504966	-0.362994
C	6	-2.5932922	0.582213	0.96008937
C	6	-4.4144699	2.65078584	1.0868505
C	6	-3.4961776	1.77662013	-1.3665198
C	6	-0.85631	2.13278873	-0.5000105
C	6	-1.8128849	3.07584039	1.97815714
C	6	0.59223632	5.89236278	-1.3376739
C	6	1.44739847	4.5495775	0.84893676
H	1	1.0137825	5.05478584	-1.9004219
H	1	0.61279122	6.8052809	-1.9382596
H	1	-0.4339868	5.66590663	-1.0278184
H	1	1.69086279	3.78873107	0.10327392
H	1	2.13973769	4.50618194	1.69314191
H	1	0.42062261	4.39547703	1.19558797

$Fe(CN)_6^{3-}$ DMSO

**ATOM**	CHARGE	X	Y	Z
FE	26	-2.6078735	2.37295945	0.37326255
S	16	1.17622246	6.34333081	-0.1435204
O	8	2.62207533	6.81932837	-0.270034
N	7	-2.3254549	5.45471777	-0.0662012
N	7	-5.2640178	2.68928379	1.99555898

N	7	-4.1908256	1.88421322	-2.2770502
N	7	0.08965457	1.93958143	-1.1531878
N	7	-1.0324706	2.83752071	3.03411833
N	7	-2.8660819	-0.7060635	0.85328005
C	6	-2.4515684	4.30085521	0.07154917
C	6	-2.7686801	0.44689084	0.68512937
C	6	-4.2705473	2.59144287	1.3874748
C	6	-3.5930276	2.08785157	-1.2933332
C	6	-0.9310549	2.12360444	-0.6148806
C	6	-1.6250901	2.64926311	2.04435358
C	6	0.7764439	5.46583238	-1.6672068
C	6	1.19526896	4.91024186	0.94953801
H	1	1.53300944	4.70037499	-1.8569314
H	1	0.76422247	6.20028898	-2.4758124
H	1	-0.2094406	5.00996749	-1.5534069
H	1	1.91733965	4.17948372	0.57566637
H	1	1.48914769	5.26022018	1.94152508
H	1	0.19482805	4.47432542	0.99084371

$Fe(CN)_6^{4-}$ ethanol

**ATOM**	CHARGE	X	Y	Z
FE	26	-0.2709894	1.28588404	-0.4059803
C	6	-0.6138973	2.98334161	0.52606296
C	6	0.02845955	-0.4190542	-1.3234591
C	6	-2.1695806	1.24604399	-0.9079853
C	6	1.61102641	1.33122321	0.09770983
C	6	-0.6159399	0.31384144	1.26514717
C	6	0.09642381	2.26572015	-2.0658442
N	7	-0.8710955	3.99664867	1.0668557
N	7	-3.2932316	1.23759673	-1.2609472
N	7	-0.7989495	-0.2936126	2.25789624
N	7	0.22110146	-1.4551575	-1.8506826
N	7	2.72943992	1.31723025	0.46925091
N	7	0.27694796	2.86233649	-3.0658592
C	6	2.2837295	5.04444139	-0.0983213
C	6	2.67614853	4.60458764	1.30307167
O	8	3.84449123	3.79263368	1.33154631
H	1	3.56551402	2.91167344	1.00209035
H	1	3.1105587	5.57227585	-0.5897223
H	1	1.41477668	5.71011971	-0.0502436
H	1	1.99185834	4.17950096	-0.7030533
H	1	1.83011718	4.0702445	1.75373377
H	1	2.89819324	5.48078689	1.92468832

$Fe(CN)_6^{3-}$ ethanol

**ATOM**	CHARGE	X	Y	Z
FE	26	-0.3137232	1.20807373	-0.3404391
C	6	-0.6261675	2.85101081	0.67570028
C	6	-0.0041705	-0.443468	-1.3476477
C	6	-2.2349575	0.9805527	-0.6693474
C	6	1.60200885	1.41090539	-0.0034643
C	6	-0.5377712	0.20800954	1.3289197
C	6	-0.0765186	2.20580584	-2.0110831
N	7	-0.8510546	3.82238657	1.28546653
N	7	-3.377287	0.86431723	-0.8897348
N	7	-0.6418504	-0.4123608	2.31419142
N	7	0.21568722	-1.4176608	-1.9549029
N	7	2.74372324	1.51716612	0.22361628
N	7	0.03697199	2.81976771	-2.9993078
C	6	2.33918701	5.12275809	-0.2625602
C	6	2.72426163	4.74754537	1.16042595
O	8	3.90492256	3.95661087	1.22554651
H	1	3.66367295	3.07845032	0.87499065
H	1	3.1641567	5.6435397	-0.763067
H	1	1.46241036	5.78038827	-0.2567141
H	1	2.079054	4.22919959	-0.8396111
H	1	1.88454118	4.22196582	1.63386017
H	1	2.93165277	5.64940604	1.747002

$Fe(CN)_6^{4-}$ acetonitrile

	CHARGE	X	Y	Z
FE	26	7.94113045	-8.9568231	-8.303334
N	7	18.8086872	-6.0502326	-14.523992
N	7	9.77930731	-3.4885135	-9.7424805
N	7	6.35701173	-6.7945868	-2.992729
N	7	2.55051509	-7.3877974	-10.210695
N	7	5.99615977	-14.416368	-6.867867
N	7	9.52743991	-11.124642	-13.584186
N	7	13.2122959	-10.502141	-6.0666323
C	6	14.8968334	-7.3715474	-11.802622
C	6	17.0746824	-6.6427228	-13.320165
C	6	6.99341538	-7.628565	-4.9551968
C	6	9.12419543	-5.5650653	-9.2828679
C	6	11.2740909	-9.9042078	-6.9842853
C	6	4.58332106	-8.0095822	-9.5525015
C	6	6.68698122	-12.353126	-7.3342372

C	6	8.88350769	-10.287661	-11.624636
H	1	13.6605474	-8.6515867	-12.855388
H	1	13.7826681	-5.7130226	-11.27105
H	1	15.5003378	-8.327962	-10.073852

$Fe(CN)_6^{3-}$ acetonitrile

**ATOM**	CHARGE	X	Y	Z
FE	26	4.19845172	-4.7781956	-4.3582426
N	7	7.00803331	-5.6192736	-3.2763916
N	7	4.93139218	-5.8202694	-7.212472
N	7	3.19723684	-7.6793039	-3.6941637
N	7	1.37563789	-3.9393095	-5.4095191
N	7	3.45478958	-3.7259876	-1.5067158
N	7	5.16190793	-1.8683174	-5.0086464
N	7	10.2449476	-3.7672517	-7.3173813
C	6	4.64559117	-5.4295573	-6.1485145
C	6	3.56223833	-6.5938858	-3.9297271
C	6	2.43661426	-4.2590591	-5.0374607
C	6	5.95591792	-5.2980427	-3.6719242
C	6	4.81462446	-2.9608174	-4.7804753
C	6	3.74329961	-4.1256247	-2.5665628
C	6	9.15881816	-3.7351852	-6.9129247
C	6	7.79495295	-3.6852939	-6.4013905
H	1	7.75954825	-4.0655153	-5.3768775
H	1	7.42797631	-2.6565688	-6.4033426
H	1	7.13455164	-4.3011595	-7.0179542

$Fe(CN)_6^{4-}$ 1-butanol

**ATOM**	CHARGE	X	Y	Z
FE	26	-3.0550671	2.2978923	0.09785898
C	6	-3.6155729	4.05930416	-0.5874728
C	6	-4.7771688	1.48082219	-0.4313004
C	6	-2.4797297	0.55122164	0.79825782
C	6	-3.8120183	2.71564163	1.86725697
C	6	-2.3248183	1.86423552	-1.6762526
C	6	-1.3306577	3.07529589	0.59597943
N	7	-3.8858412	5.13687856	-0.9755373
N	7	-5.8000504	1.00463446	-0.7698063
N	7	-4.321099	2.91685601	2.90986651
N	7	-2.1537986	-0.5063639	1.20083594
N	7	-0.2818041	3.52044042	0.89557582
N	7	-1.8611892	1.64792193	-2.7376585
C	6	-3.8304735	7.43412476	2.17121291

C	6	-3.2979267	6.44791795	3.21163907
C	6	-1.8667019	6.0081194	2.90984293
C	6	-1.3817894	4.90192961	3.83808157
O	8	-0.033739	4.52605358	3.57976489
H	1	-4.8602853	7.7376665	2.39501028
H	1	-3.817277	6.97144941	1.17690604
H	1	-3.214141	8.3423665	2.13666276
H	1	-3.3483655	6.89527467	4.21562245
H	1	-3.9317427	5.55302407	3.2239262
H	1	-1.1803505	6.86539294	2.97805692
H	1	-1.825054	5.62790704	1.88155104
H	1	-2.0513746	4.0380455	3.73418347
H	1	-1.4145275	5.23503009	4.88350831
H	1	-0.0217522	4.17764222	2.66254163

$Fe(CN)_6^{3-}$ 1-butanol

**ATOM**	CHARGE	X	Y	Z
FE	26	-3.1973921	1.84767933	0.05897958
O	8	0.14746195	4.93225432	3.4345121
N	7	-1.6074946	2.2529957	-2.6058911
N	7	-0.690953	2.98347314	1.53418836
N	7	-2.1068648	-1.0861074	0.15351929
N	7	-4.7859871	1.43064423	2.71989783
N	7	-5.7519649	0.83526545	-1.4316292
N	7	-4.3094545	4.77103974	-0.0510517
C	6	-1.0909151	5.46115061	3.88021705
C	6	-1.8213588	6.26616083	2.81033665
C	6	-3.1246403	6.87451345	3.32949064
C	6	-3.8559836	7.69065632	2.26377361
C	6	-1.6293584	2.54995243	0.99043421
C	6	-2.2154627	2.09846202	-1.6195085
C	6	-4.1780334	1.59187426	1.73462525
C	6	-2.5092551	0.01062983	0.11128948
C	6	-4.7830359	1.18006418	-0.8757343
C	6	-3.8908599	3.68130743	0.00393361
H	1	-0.0603208	4.25718149	2.75677675
H	1	-0.8596163	6.10469852	4.73760686
H	1	-1.7442476	4.65258273	4.24298379
H	1	-2.0405009	5.61462285	1.95400974
H	1	-1.1541862	7.06363911	2.45361041
H	1	-3.7822873	6.06880092	3.68331489
H	1	-2.9117319	7.50888616	4.20186314
H	1	-3.2329194	8.52304441	1.91296495

H	1	-4.1012695	7.05904176	1.40243396
H	1	-4.7888592	8.11252028	2.65601344

$Fe(CN)_6^{4-}$ water

**ATOM**	CHARGE	X	Y	Z
FE	26	-0.8942679	0.65837861	0.11234439
O	8	-0.7941141	-0.7290405	3.97623542
N	7	0.3086665	3.23210551	-1.2206714
N	7	1.41944552	0.60895562	2.21371572
N	7	0.77705156	-1.2899461	-1.7105595
N	7	-2.1703147	-1.8553439	1.45400849
N	7	-3.1961645	0.74483409	-2.0209621
N	7	-2.5918926	2.64106244	1.86621143
C	6	0.55052461	0.6431041	1.41810952
C	6	-0.1149969	2.24667165	-0.7342005
C	6	0.16499046	-0.5403482	-1.0397305
C	6	-1.7074771	-0.9076528	0.92760646
C	6	-2.3273157	0.70637826	-1.2273662
C	6	-1.9554874	1.85702016	1.2619819
H	1	-1.3739652	-1.0327096	3.25617982
H	1	-0.1369153	-0.2124315	3.47875175

$Fe(CN)_6^{3-}$ water

**ATOM**	CHARGE	X	Y	Z
FE	26	-1.0347346	0.82655884	0.08017258
O	8	-0.0806207	-1.6254794	3.60682454
N	7	0.5257203	2.94147778	-1.6119451
N	7	1.07783279	1.02315543	2.37057637
N	7	0.71666733	-1.3921284	-1.2591425
N	7	-2.5937845	-1.2567932	1.80671449
N	7	-3.130795	0.61175591	-2.2282491
N	7	-2.7751854	3.06366297	1.40456405
C	6	0.28431735	0.92269941	1.51765544
C	6	-0.0666984	2.1519527	-0.9859758
C	6	0.0754475	-0.5580519	-0.7504191
C	6	-2.0015596	-0.4759626	1.16866531
C	6	-2.3501347	0.70576107	-1.3635567
C	6	-2.1367477	2.22314757	0.90235673
H	1	-0.8690216	-1.6698841	3.04271324
H	1	0.37738643	-0.8431557	3.26093275

acetone

ATOM	CHARGE	X	Y	Z

O	8	-4.081859	3.07358426	0.29256513
C	6	-3.3425326	0.82989304	-0.0597203
C	6	-4.3880278	1.90781913	0.09605519
C	6	-5.8298021	1.47213652	0.00010691
H	1	-3.31335	0.50838625	-1.1079355
H	1	-2.3579189	1.20461516	0.22599035
H	1	-3.6013817	-0.0499419	0.5387983
H	1	-5.9893995	0.86178202	-0.895736
H	1	-6.0716444	0.84185055	0.86506186
H	1	-6.4939124	2.3384167	-0.0129527

DMSO

ATOM	CHARGE	X	Y	Z
C	6	0.61004438	5.4628867	-1.3707886
H	1	1.62445237	5.40362684	-1.7760525
H	1	0.06335902	6.27141067	-1.8607997
S	16	0.6768367	5.83674452	0.39539808
H	1	0.07951194	4.51821903	-1.5173966
C	6	1.69182807	4.4232522	0.88148869
H	1	2.64102084	4.44543761	0.33759486
H	1	1.87726244	4.50489831	1.95485082
H	1	1.14711481	3.49913847	0.67088226
O	8	1.53574191	7.07956991	0.55853328

ethanol

ATOM	CHARGE	X	Y	Z
O	8	-1.4791711	1.02477667	-0.3892225
C	6	-2.0392336	-0.1645988	0.15407872
C	6	-3.5456596	-0.112264	-0.0076237
H	1	-0.5218603	0.97842656	-0.2741135
H	1	-1.643139	-1.048801	-0.3650296
H	1	-1.7859413	-0.2584523	1.21949307
H	1	-3.817535	-0.0338922	-1.0657612
H	1	-4.0007424	-1.0212983	0.39942274
H	1	-3.9630331	0.75074251	0.52214045

acetonitrile

ATOM	CHARGE	X	Y	Z
N	7	-4.6697074	1.00787991	-0.0007897
C	6	-5.8271804	1.05944148	0.00089516
C	6	-7.2852103	1.12390841	0.00051527
H	1	-7.6977487	0.1492983	-0.2721113
H	1	-7.6417121	1.40263909	0.99557067

H	1	-7.6206123	1.87055451	-0.7240795

1-butanol

**ATOM**	CHARGE	X	Y	Z
C	6	-3.7549607	7.53104042	1.98912282
C	6	-2.9766251	6.98399938	3.1852002
C	6	-1.742467	6.18244017	2.76744474
C	6	-0.9556691	5.65687115	3.96081259
O	8	0.19098498	4.90514836	3.57621081
H	1	-4.6400239	8.09024886	2.3121032
H	1	-4.0908318	6.71734184	1.33490893
H	1	-3.1303807	8.20559648	1.39103406
H	1	-2.6696014	7.81680294	3.83236673
H	1	-3.6359534	6.34667801	3.79017971
H	1	-1.079215	6.80639331	2.15396169
H	1	-2.0546701	5.33473479	2.13970119
H	1	-1.607427	5.05648188	4.61258531
H	1	-0.574752	6.49335143	4.55516857
H	1	-0.1165631	4.10628665	3.12734429

water

**ATOM**	CHARGE	X	Y	Z
O	8	-3.2491695	0.11845519	-0.0196314
H	1	-3.5218504	-0.1923942	0.85226572
H	1	-2.2853964	0.10119454	0.02878276

$Fe(CN)_6^{4-}$ in acetone

ATOM	CHARGE	X	Y	Z
FE	26	-0.6753432	1.01216456	-0.0821347
N	7	-3.1829751	1.63250137	-1.9146324
N	7	1.94899682	0.67977122	1.65024633
N	7	1.2299181	2.81564682	-1.842426
N	7	0.24555879	-1.4587401	-1.7777589
N	7	-2.3151067	-0.9600032	1.7732479
N	7	-1.440511	3.47946969	1.6934636
C	6	0.48936416	2.15550107	-1.2078811
C	6	0.95468761	0.78343207	1.02814333
C	6	-0.1290746	-0.5365286	-1.1485179
C	6	-1.7219152	-0.2027944	1.09289532
C	6	-2.250997	1.37148469	-1.242936

C	6	-1.1566691	2.56542662	1.00668811

$Fe(CN)_6^{3-}$ in acetone

ATOM	CHARGE	X	Y	Z
FE	26	-0.6190584	1.02589489	-0.073133
N	7	-1.3310142	3.52921728	1.66201588
N	7	-2.3284986	-0.9089393	1.70555774
N	7	0.08823328	-1.483147	-1.8039208
N	7	1.08601396	2.96378466	-1.8521487
N	7	1.83777503	0.56987227	1.81569751
N	7	-3.073389	1.4705618	-1.966055
C	6	-1.0723674	2.59198908	1.01306114
C	6	-2.1423915	1.31898409	-1.2751531
C	6	-1.6974416	-0.176628	1.04794447
C	6	-0.1670814	-0.5426629	-1.1584028
C	6	0.90585931	0.72807061	1.12764836
C	6	0.45620931	2.23058388	-1.194297

$Fe(CN)_6^{4-}$ in DMSO

ATOM	CHARGE	X	Y	Z
FE	26	-1.2782222	1.90793115	-0.1600925
N	7	-5.9681012	3.13104533	-3.6605836
N	7	3.64442421	1.24020664	3.16445971
N	7	2.3578396	5.34808592	-3.3906492
N	7	0.50249403	-2.7429482	-3.374799
N	7	-4.4384489	-1.8176526	3.27450284
N	7	-2.7556822	6.544077	3.20874998
C	6	0.95382369	4.07779669	-2.2216415
C	6	1.77056566	1.43903274	1.98123294
C	6	-0.2096993	-1.0015553	-2.186352
C	6	-3.3027042	-0.3834097	2.00678001
C	6	-4.2171893	2.6272208	-2.3816457
C	6	-2.2168178	4.81869461	1.90963786

$Fe(CN)_6^{3-}$ in DMSO

ATOM	CHARGE	X	Y	Z
FE	26	-1.1894459	1.95483284	-0.1434987
N	7	-2.9610341	6.54720243	3.131599
N	7	-4.3432416	-1.6355187	3.33399277
N	7	0.54757267	-2.6468386	-3.4280547
N	7	2.21510522	5.36609009	-3.5710317
N	7	3.52122591	0.84791643	3.27731049
N	7	-5.886906	3.07158195	-3.5795745

C	6	-2.2920931	4.82645214	1.9190501
C	6	-4.1198114	2.64304242	-2.3249348
C	6	-3.187046	-0.250606	2.05809105
C	6	-0.1066417	-0.9264431	-2.2066587
C	6	1.75206344	1.27936155	2.02619595
C	6	0.89253449	4.11145167	-2.3228863

$Fe(CN)_6^{4-}$ in ethanol

ATOM	CHARGE	X	Y	Z
FE	26	-1.2735974	1.90975796	-0.1583601
N	7	-5.9681862	3.12498388	-3.6621689
N	7	3.64663702	1.23577888	3.17067874
N	7	2.36106067	5.34598736	-3.4017283
N	7	0.49815261	-2.7404461	-3.3792214
N	7	-4.438224	-1.8150523	3.27499588
N	7	-2.7668965	6.5445606	3.21314423
C	6	0.95848037	4.07893173	-2.2272257
C	6	1.77298574	1.43742838	1.98707684
C	6	-0.2098673	-1.0001238	-2.1868051
C	6	-3.3009057	-0.3808451	2.00811068
C	6	-4.2169622	2.62479742	-2.3817542
C	6	-2.220395	4.82276636	1.91285743

$Fe(CN)_6^{3-}$ in ethanol

ATOM	CHARGE	X	Y	Z
FE	26	-1.1898804	1.95383247	-0.1437197
N	7	-2.9513034	6.54733259	3.13676845
N	7	-4.3501027	-1.6367333	3.33108159
N	7	0.55282244	-2.6483547	-3.4272841
N	7	2.21181917	5.36698995	-3.5732683
N	7	3.5211606	0.85451231	3.28009951
N	7	-5.887948	3.07306564	-3.5814657
C	6	-2.2881244	4.82617854	1.92158199
C	6	-4.1225818	2.64128749	-2.3249541
C	6	-3.1906594	-0.2523137	2.05730637
C	6	-0.1039821	-0.9278079	-2.2076511
C	6	1.75166688	1.27912365	2.02710559
C	6	0.88939527	4.1114121	-2.3260006

$Fe(CN)_6^{4-}$ in acetonitrile

ATOM	CHARGE	X	Y	Z
FE	26	-1.2750428	1.90879135	-0.1598349
N	7	-5.9670044	3.12978929	-3.6616034

N	7	3.64512938	1.24064003	3.16569723
N	7	2.3589687	5.34812714	-3.3926469
N	7	0.49952606	-2.7408076	-3.3782203
N	7	-4.4409226	-1.8194761	3.27767875
N	7	-2.7604275	6.54313942	3.20961415
C	6	0.95621282	4.07771126	-2.2227237
C	6	1.7723415	1.43871371	1.98089968
C	6	-0.2101656	-1.0001157	-2.1875286
C	6	-3.3037192	-0.3853553	2.01088556
C	6	-4.2159375	2.62698546	-2.3819234
C	6	-2.2166769	4.82038229	1.90930564

$Fe(CN)_6^{3-}$ in acetonitrile

ATOM	CHARGE	X	Y	Z
FE	26	-1.189796	1.9541374	-0.1433185
N	7	-2.9613679	6.54685169	3.13200313
N	7	-4.3415805	-1.6375301	3.33488122
N	7	0.54294039	-2.647876	-3.4296938
N	7	2.21861087	5.36593742	-3.5681197
N	7	3.5229636	0.85405369	3.27611721
N	7	-5.8862824	3.07463262	-3.5820676
C	6	-2.2925829	4.82556367	1.92000641
C	6	-4.1214708	2.64079866	-2.3255372
C	6	-3.1864217	-0.2514635	2.05904606
C	6	-0.1080099	-0.9271782	-2.2073034
C	6	1.75169567	1.27901362	2.02580085
C	6	0.89358358	4.11158424	-2.3222147

$Fe(CN)_6^{4-}$ in 1-butanol

ATOM	CHARGE	X	Y	Z
FE	26	-1.1710402	1.93595905	-0.1410208
N	7	-5.9454608	3.05831729	-3.5971737
N	7	3.60886723	0.8405864	3.29735786
N	7	2.1152603	5.55874366	-3.528116
N	7	0.53978939	-2.7365829	-3.36485
N	7	-4.421371	-1.7019436	3.26663124
N	7	-2.8797409	6.60966737	3.08288703
C	6	0.94114529	4.15354027	-2.2610291
C	6	1.82413093	1.29412062	2.04736944
C	6	-0.1557515	-0.9924344	-2.1705758
C	6	-3.2619591	-0.2900883	1.9936188
C	6	-4.1661171	2.59328608	-2.3434594
C	6	-2.1854703	4.86535365	1.88796031

$Fe(CN)_6^{3-}$ in 1-butanol

ATOM	CHARGE	X	Y	Z
FE	26	-0.6315457	1.02993686	-0.0765125
N	7	-3.1571748	1.57312732	-1.8589907
N	7	1.90744972	0.51913033	1.69266835
N	7	1.21471086	2.88982916	-1.7927251
N	7	0.24380273	-1.4424976	-1.7833466
N	7	-2.3462054	-0.9135864	1.68130945
N	7	-1.5102227	3.50435654	1.62354696
C	6	0.50386513	2.20954843	-1.1614331
C	6	0.95797251	0.71520846	1.03959643
C	6	-0.0949035	-0.5294242	-1.136945
C	6	-1.7247506	-0.1737035	1.02292995
C	6	-2.216387	1.3567893	-1.1991606
C	6	-1.1677307	2.59047978	0.98045636

$Fe(CN)_6^{4-}$ in water

ATOM	CHARGE	X	Y	Z
FE	26	-1.2775971	1.90932445	-0.158707
N	7	-5.9679241	3.13105953	-3.6590044
N	7	3.64379681	1.24146559	3.16647166
N	7	2.36088415	5.34286248	-3.3943275
N	7	0.49858971	-2.7398131	-3.3752284
N	7	-4.4350514	-1.8196058	3.27135999
N	7	-2.7604582	6.54234513	3.20958233
C	6	0.9560177	4.07575668	-2.2232897
C	6	1.77022699	1.44017459	1.98323109
C	6	-0.2120051	-0.9991333	-2.1852956
C	6	-3.2998496	-0.382917	2.00573559
C	6	-4.2165662	2.62792348	-2.3804145
C	6	-2.2177815	4.81908242	1.90948631

$Fe(CN)_6^{3-}$ in water

ATOM	CHARGE	X	Y	Z
FE	26	-1.190643	1.95405585	-0.1441954
N	7	-2.963639	6.54547629	3.13070728
N	7	-4.3390066	-1.6372498	3.33539883
N	7	0.54538605	-2.6470835	-3.4281952
N	7	2.22146633	5.36416759	-3.5653611
N	7	3.52133397	0.85386817	3.27486156
N	7	-5.8870345	3.07692334	-3.5811235
C	6	-2.2936472	4.82488177	1.91836862

C	6	-4.1222764	2.64095257	-2.3251217
C	6	-3.1849735	-0.2501573	2.06009527
C	6	-0.1098465	-0.9269508	-2.2076299
C	6	1.75112915	1.27932032	2.02348504
C	6	0.89403317	4.11032063	-2.32169

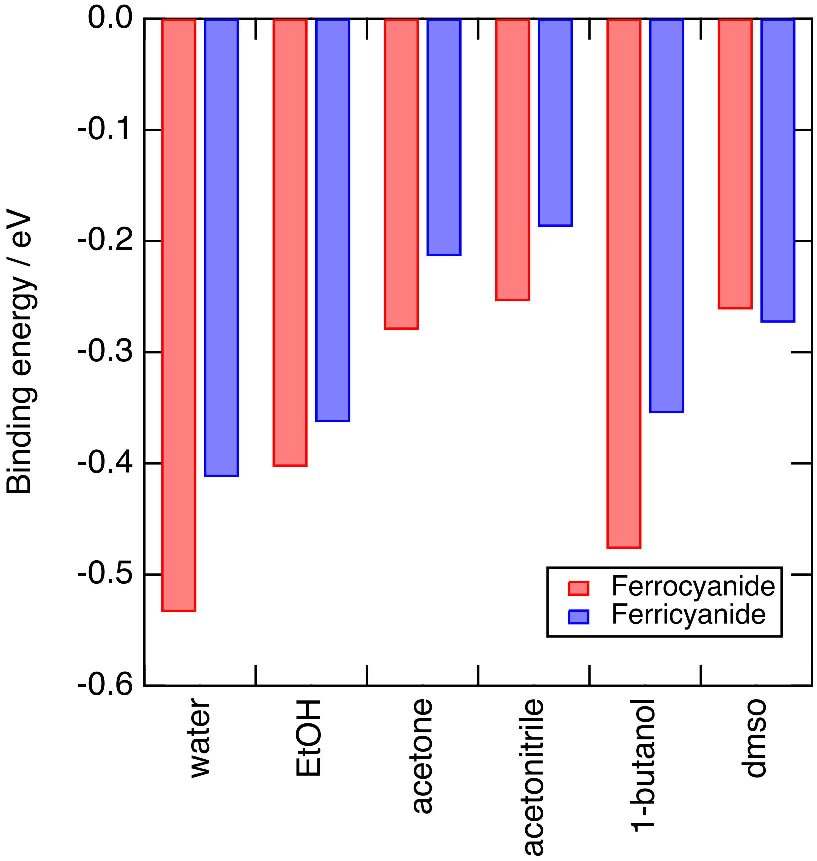

Figure S3. The calculated binding energy between ferrocyanide/ferrycyanide and the candidate solvents.

Table. S1 The closest distance between the N atom of ferrocyanide/ferricyanide and the H atom of methyl group of the solvents.

	Distance between N and H / Å	
	Acetone	DMSO
Ferrocyanide	2.31	2.51
Ferricyanide	2.62	2.53

Table. S2 The distance between ferrocyanide/ferricyanide and the solvents.

	Acetone	DMSO
Ferrocyanide	5.28	5.67
Ferricyanide	5.27	5.51

Analysis of the reversibility of the redox system from CV

To assess the reversibility of ferrocyanide/ferricyanide in the electrolyte with x_s = 0.05 and 0.9, CVs with its scan rate 100, 50, 10, 1 mV s^{-1} were measured. Note that CVs with its scan rate 1 mV^{-1} in water-acetone system are omitted since the current peak are too small to determine them. From the CV curves, peak separation (ΔE_p) was summarized in Table S2–S4. The reversible redox system shows $\Delta E_p = 0.057/n$ V, where n is the number of electrons in the redox reaction. Figure S4 and S5 shows the linear relationship between the peak current and square root of the scan rate. ΔE_p were over 0.1 V. This is derived from the high solution resistance as shown in Figure 5(b).

The diffusion coefficients (D_0) of ferrocyanide /ferricyanide were calculated using the following Equation (S1).

$$I_p = (0.4463) nFAc_0 \sqrt{\frac{nFvD_0}{RT}} \tag{S1}$$

where A, c_0, I_p, and v denote the electrode area, bulk concentration of ferrocyanide/ferricyanide, peak current, and scan rate, respectively.

Table S3. The anolyte and catholyte composition of water-acetone system we have previously reported.

	Anolyte	Catholyte
x_s	0.14	0
Ferrocyanide / mol kg^{-1}	0.24	0.040
Ferricyanide / mol kg^{-1}	0.026	0.36

Table S4. Electrochemical parameters of ferrocyanide/ferricyanide in water-DMSO mixed electrolyte of x_s = 0.05.

Scan rate / mV s^{-1}	100	50	10	1
ΔE_p / V	0.365	0.307	0.199	0.184

Table S5. Electrochemical parameters of ferrocyanide/ferricyanide in water-DMSO mixed electrolyte of x_s = 0.9.

Scan rate / mV s^{-1}	100	50	10	1
ΔE_p / V	0.561	0.459	0.285	0.150

Table S6. Electrochemical parameters of ferrocyanide/ferricyanide in anolyte (x_s = 0.14) of water-acetone system.

Scan rate / mV s^{-1}	100	50	10
ΔE_p / V	0.303	0.244	0.180

Table S7. Electrochemical parameters of ferrocyanide/ferricyanide in catholyte (x_s = 0) of water-acetone system.

Scan rate / mV s^{-1}	100	50	10
ΔE_p / V	0.236	0.192	0.127

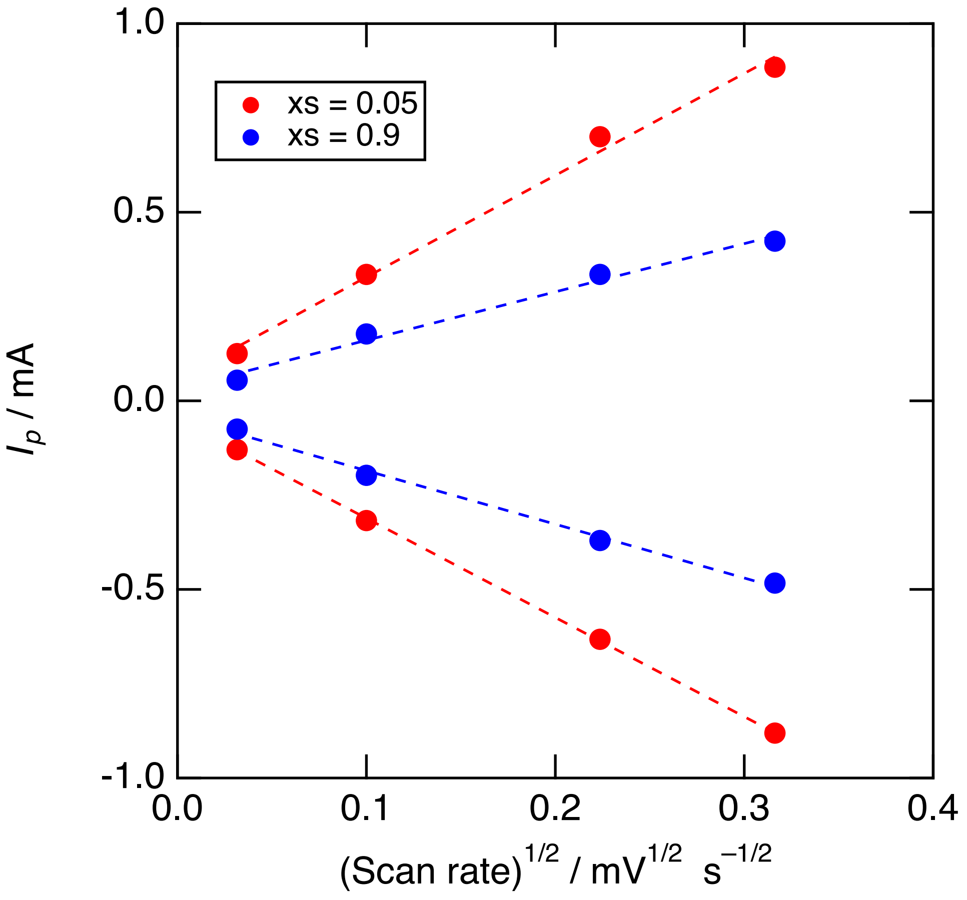

Figure S4. The relationship between the peak current and the square root of the scan rate in water-DMSO system. The dotted lines indicate the fitting lines.

Table S8. The coefficient of determination (R^2) of the linear fitting of Figure S4.

x_s		R^2
0.05	I_{pa}	0.9927
	I_{pc}	0.9999
0.9	I_{pa}	0.9866
	I_{pc}	0.9948

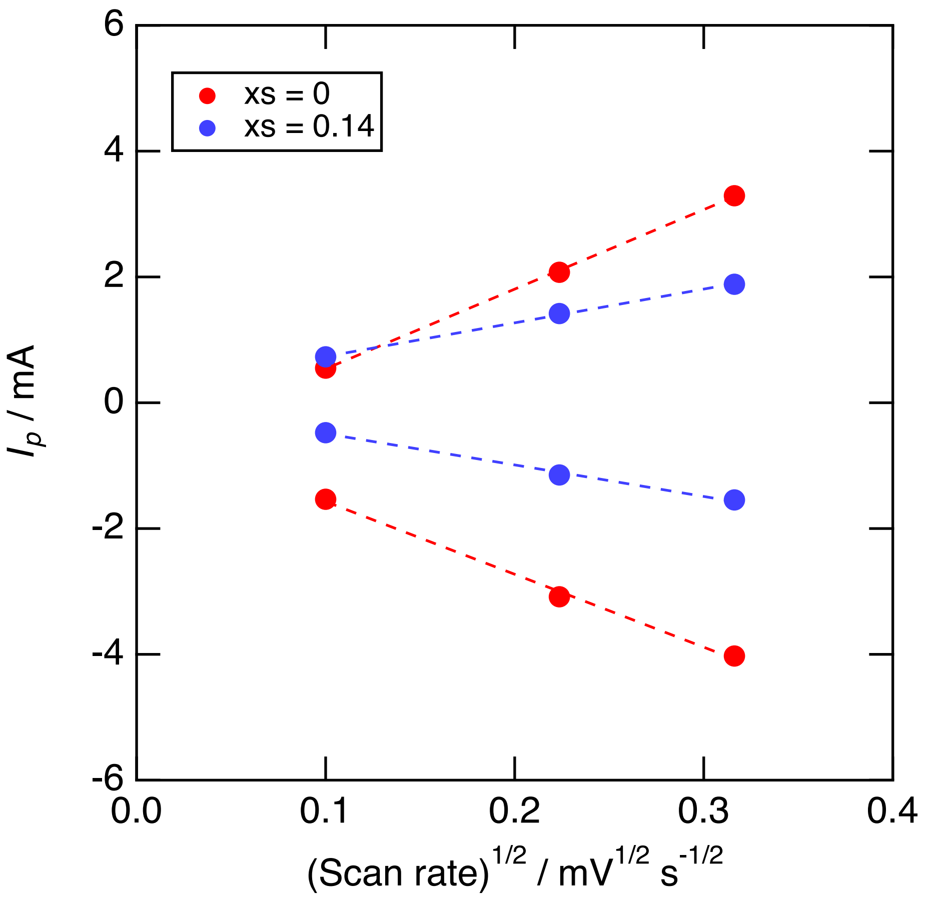

Figure S5. The relationship between the peak current and the square root of the scan rate in water-acetone system. The dotted lines indicate the fitting lines.

Table S9. The coefficient of determination (R^2) of the linear fitting of Figure S5.

x_s		R^2
0	I_{pa}	0.9997
	I_{pc}	0.9966
0.14	I_{pa}	0.9993
	I_{pc}	0.9959

To determine the standard rate constant (k_0), plots of $\ln(I_p)$ vs. $(E_p - E^0)$ are shown in Figure S6 and S7. E^0 indicates the formal potential. The following equation is established between $\ln(I_p)$ and $(E_p - E^0)$.

$$\ln(I_p) = \ln(0.227FAk^0C) - (\alpha F/RT)(E_p - E^0)$$

$F, A, C, \alpha, R, and\ T$ means Faraday constant, surface area of the electrode, bulk concentration, charge-transfer coefficient, gas constant, and temperature. k_0 and α are determined from the slope and intercept of the plots of $\ln(I_p)$ vs. $(E_p - E^0)$. When the redox reaction is the quasi-reversible, k_0 is between 10^{-1} and 10^{-5} cm s^{-1}.[1] Table S8 and S9 mean that the redox reactions of ferrocyanide/ferricyanide in x_s = 0.05 and 0.90 are quasi-reversible due to $k_0 > 10^{-4}$ cm s^{-1}.

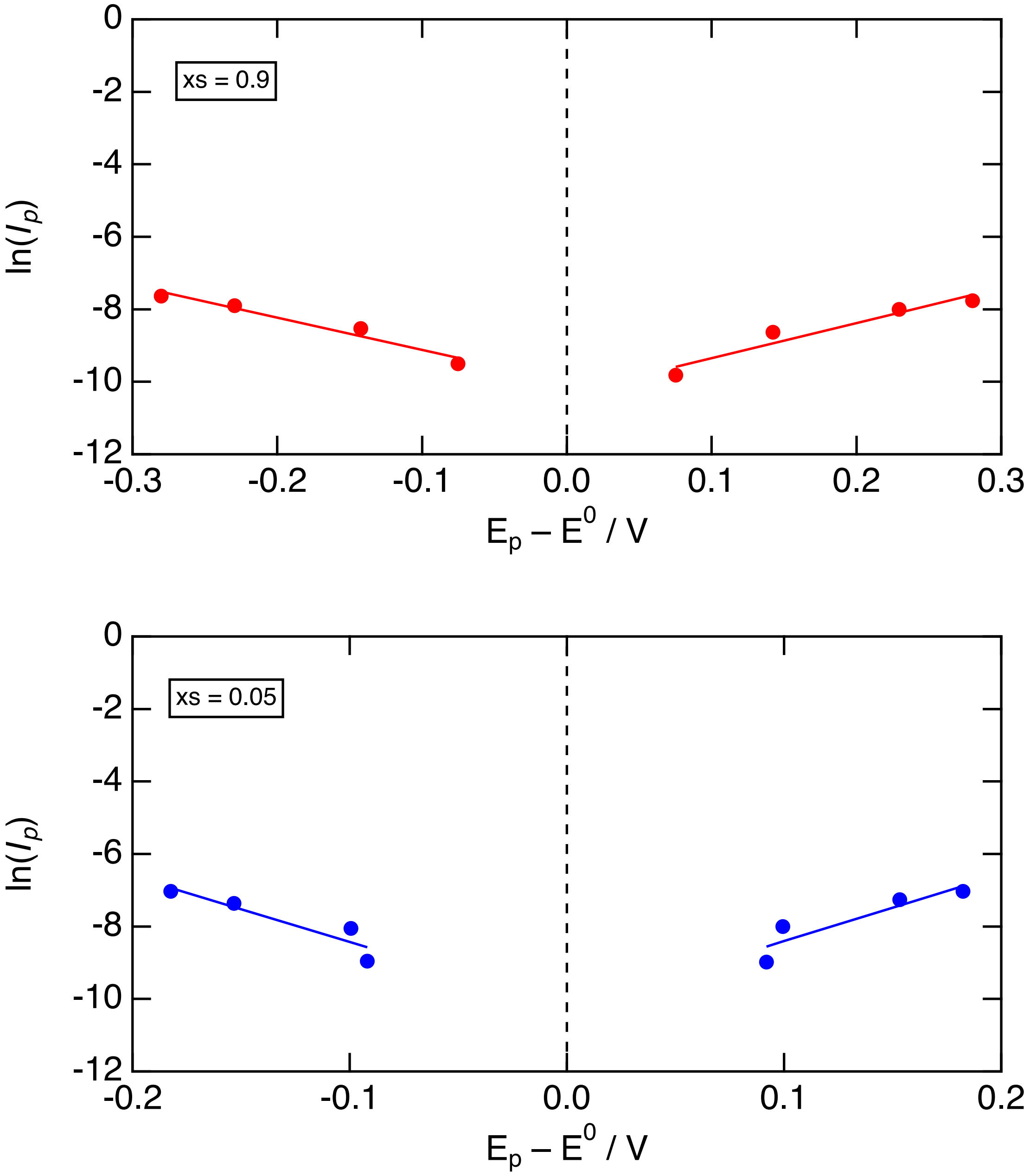

Figure S6. Plots of ln(i_p) vs. ($E_p - E^0$) in water-DMSO mixed solvents of x_s = 0.9 and 0.05.

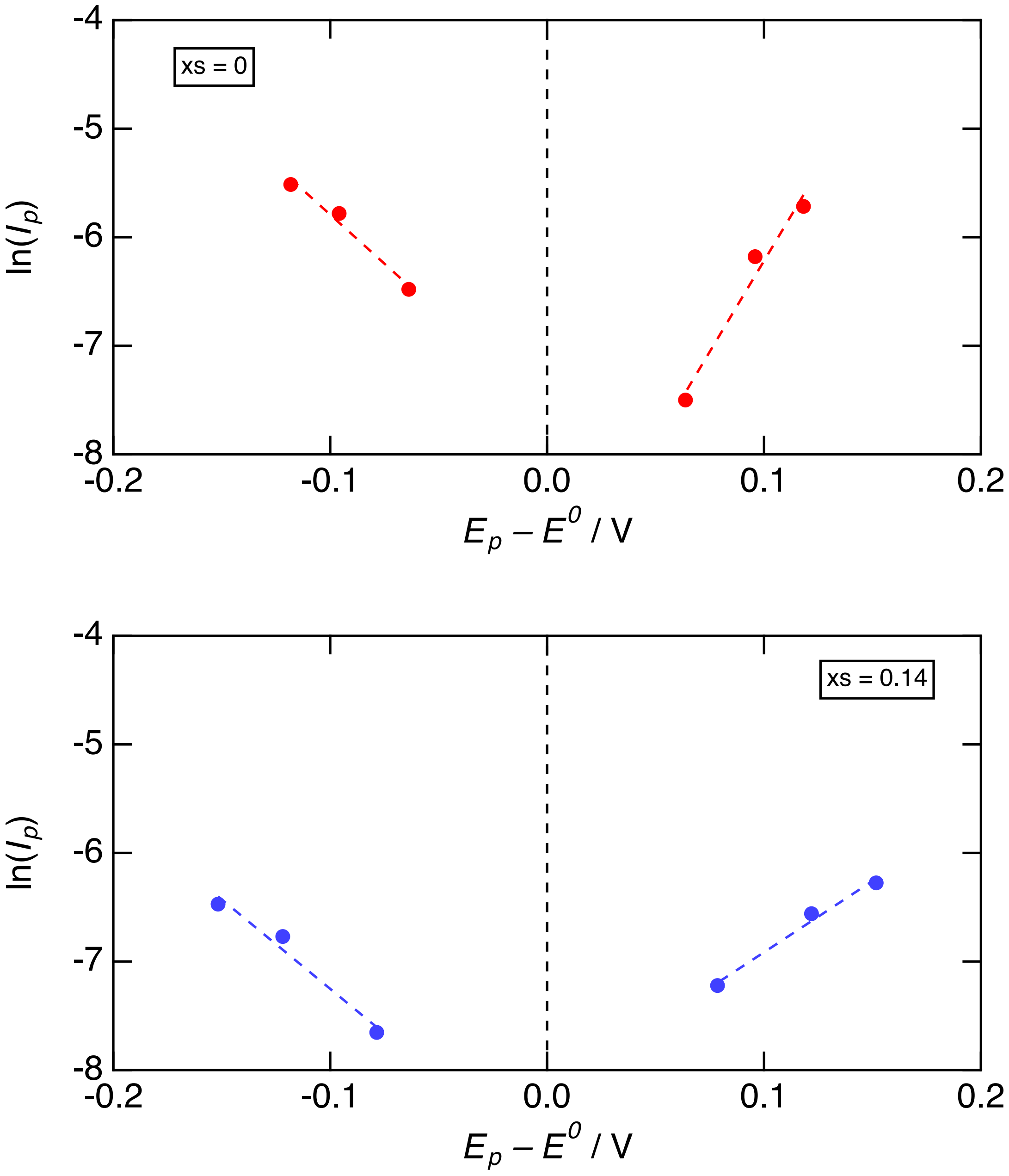

Figure S7. Plots of ln(i_p) vs. (E_p – E^0) in the anolyte and catholyte of water-acetone system. The anolyte and catholyte are in the water-acetone mixed solvents of x_s = 0.14 and 0, respectively.

Table S10. The standard rate constant analysis from CVs in water-DMSO system.				
x_s	0.05		0.90	
	anodic	cathodic	anodic	cathodic
slope	18.427	–18.1	9.6996	–8.9044
intercept	–10.248	–10.237	–10.32	–10.011
R^2	0.8266	0.8573	0.9295	0.9564
k^0 / cm s^{-1}	2.29×10^{-4}	2.31×10^{-4}	2.12×10^{-4}	2.90×10^{-4}
α	0.47	0.46	0.25	0.23

Table S11. The standard rate constant analysis from CVs in water-acetone system.				
x_s	0		0.14	
	anodic	cathodic	anodic	cathodic
slope	33.372	-18.002	13.135	-16.481
intercept	-9.5541	-7.5916	-8.2277	-8.9001
R^2	0.9719	0.9777	0.9852	0.9700
k^0 / cm s^{-1}	1.27×10^{-4}	9.05×10^{-4}	7.19×10^{-4}	3.67×10^{-4}
α	0.85	0.46	0.34	0.17

Electrochemical impedance spectroscopy of ferrocyanide/ferricyanide in water-DMSO solution

The Nyquist plots of ferrocyanide/ferricyanide in the electrolyte with x_s = 0.05 and 0.9 of water-DMSO system as well as the anolyte and catholyte of water-acetone system (Figure 5(b)) were fitted with Zview® 4 software (Scribner). In the case of x_s = 0.05 in water-DMSO system and the water-acetone system, since the semicircle and linear region were observed in the Nyquist plot, the equivalent circuit which contains R_s, R_{ct}, Z_w, and CPE was used for fitting (Figure S8(a)). On the other hand, since only the linear region was observed in x_s = 0.9, the equivalent circuit which contains R_s and Z_w was used for fitting (Figure S8(b)). This result suggests that the charge-transfer resistance in x_s = 0.9 is effectively smaller than that in x_s = 0.05.

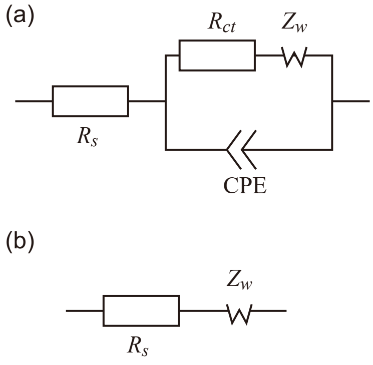

Figure S8. The used equivalent circuits for fitting of the Nyquist plots of (a) x_s = 0.05 in water-DMSO system and the water-acetone system, as well as (b) x_s = 0.9 in the water-DMSO system.

R_s, and R_{ct} are solution resistance and charge-transfer resistance. Warburg impedance (Z_w) and constant phase element (CPE) are described in Equations S2 and S3, respectively.

$$Z_w = R\frac{\tanh((jT\omega)^P)}{(jT\omega)^P} \quad \text{(S2)}$$

$$\text{CPE} = \frac{1}{T(j\omega)^P} \quad \text{(S3)}$$

The calculated parameters were summarized in Tables S10 and S11.

Table S12. The calculated parameters of the fitting of the Nyquist plots in water-DMSO mixed solvents of x_s = 0.05 and 0.9. χ^2 values of the fitting results of x_s = 0.05 and 0.9 were 1.0588×10^{-5} and 1.1843×10^{-5}.

x_s	0.05	0.9
R_s / Ω	100.2	406.1
R_{ct} / Ω	34.57	–
Z_w-R	623.7	1062
Z_w-T	98.71	82.49
Z_w-P	0.46242	0.44939
CPE-T / Ω^{-1} s^P	1.571×10^{-6}	–
CPE-P	0.94854	–

Table S13. The calculated parameters of the fitting of the Nyquist plots in water-acetone mixed solvents of x_s = 0 and 0.14. χ^2 values of the fitting results of x_s = 0 and 0.14 were 1.6788×10^{-6} and 1.7886×10^{-5}.

x_s	0	0.14
R_s / Ω	16.63	47.51
R_{ct} / Ω	8.141	3.896
Z_w-R	224.7	138.9
Z_w-T	27.68	7.452
Z_w-P	0.4918	0.4677
CPE-T / Ω^{-1} s^P	4.501×10^{-6}	1.247×10^{-6}
CPE-P	0.93426	1.014

Thermal regeneration and discharging properties of water-DMSO SDFB

The discharge test at a constant current density of 50 mA cm^{-2} was conducted until the discharge voltage decreased to 0 mV. Subsequently, the discharge tests at constant current densities of 45, 40, 35, 30, 25, 20, 10, and 5 mA cm^{-2} were also conducted for complete discharging. The initial electrolyte composition is described in Table 2, which is defined as the fully charged condition (state of charge (SoC) 100%). After the discharge, water in the catholyte was evaporated with an evaporator at 60 ºC, which is used as a regenerated anolyte. In addition, 23.41 g of water, which is the theoretical amount of water in the catholyte as shown in Table 2, was added to the anolyte, which is used as a regenerated catholyte.

Figure S9 shows the normalized capacity as a function of cycle number. The normalized capacity is defined as the capacity of each cycle divided by the theoretical capacity (40.20 mAh). Figure S9 indicates that only 60 % of the theoretical capacity is obtained even in the 1st cycle. Since the electrolyte was not completely discharged in 1st cycle, the concentration of ferrocyanide/ferricyanide was not completely regenerated even after the solvent separation. Therefore, the normalized capacity after 2nd cycle is lower than that in 1st cycle. On the other hand, the normalized capacity is stable after 2nd cycle, which suggests that the capacity loss derived from the deterioration of the electrolyte such as the deactivation of the redox species is negligible.

The crossover of the solvents and ferrocyanide/ferricyanide and the difficulty of the complete water separation from DMSO should be a reason for the capacity loss, but the details are still unclear. Further investigation and optimization of the discharging and regeneration process should be required in future work.

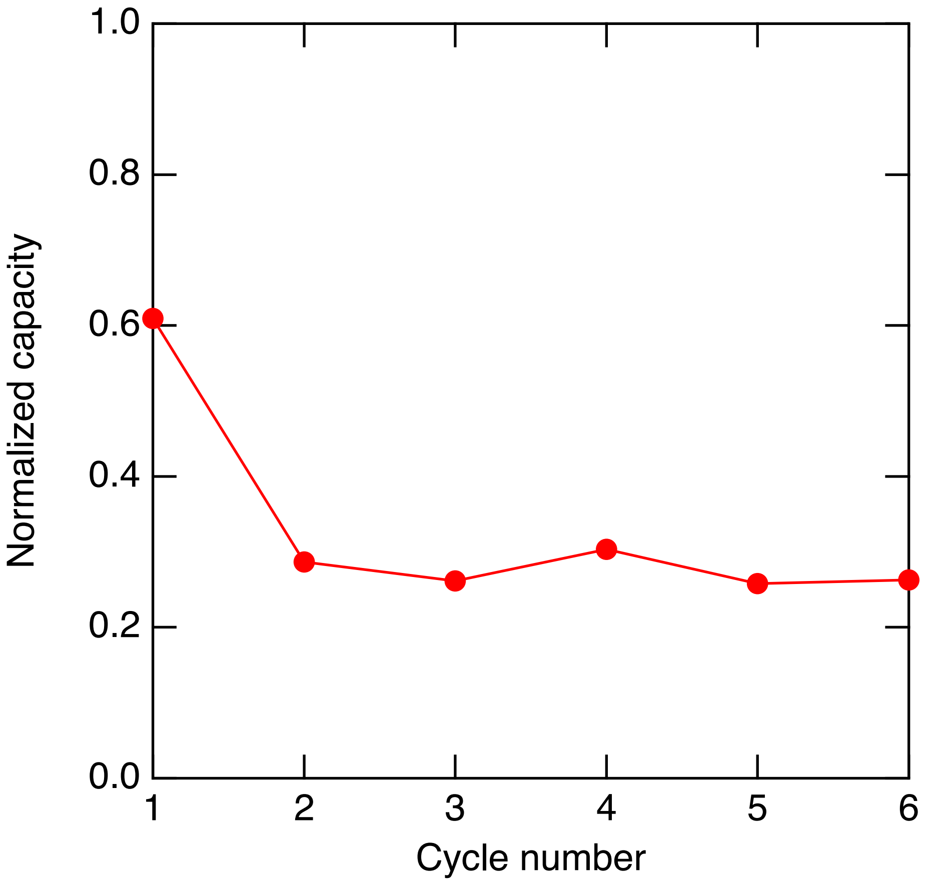

Figure S9. The normalized capacity with respect to the cycle number. The normalized capacity is defined as the capacity of each cycle divided by the theoretical capacity (40.20 mAh).

To discuss the contribution of the concentration in the electrolyte, the potential shift derived from the concentration difference (ΔE_{conc}) after the electrolyte regeneration was calculated based on Nernst equation as shown below.

The redox potential of the anolyte and catholyte is described by Nernst equation.

$$E_{anolyte} = E^{\circ}_{anolyte} - \frac{RT}{F}\ln(\frac{c_{II}(anolyte)}{c_{III}(anolyte)})$$

$$E_{catholyte} = E^{\circ}_{catholyte} - \frac{RT}{F}\ln(\frac{c_{II}(cathoylte)}{c_{III}(catholyte)})$$

where E^{o}, c_{II}, c_{III} are the standard redox potential, concentration of ferrocyanide and ferricyanide, respectively. Thus, the potential shift (ΔE) is described below.

$$\Delta E = E^{\circ}_{catholyte} - E^{\circ}_{anolyte} - \frac{RT}{F}\ln(\frac{c_{II}(catholyte)c_{III}(anolyte)}{c_{III}(catholyte)c_{II}(anolyte)})$$

In SDFB, the amount of ferrocyanide (m_{II}) /ferricyanide (m_{III}) in the anolyte and catholyte are related as follows.

$$m_{III}(catholyte) = m_{II}(anolyte)$$

$$m_{II}(catholyte) = m_{III}(anolyte)$$

Therefore, ΔE is also described below.

$$\Delta E = E^{\circ}_{catholyte} - E^{\circ}_{anolyte} - \frac{2RT}{F}\ln(\frac{m_{III}(anolyte)}{m_{II}(anolyte)})$$

In the above equation, $E^{\circ}_{catholyte} - E^{\circ}_{anolyte}$ is derived from the solvation difference, and $-\frac{2RT}{F}\ln\left(\frac{m_{III}(anolyte)}{m_{II}(anolyte)}\right) = \Delta E_{conc}$ is derived from the concentration difference.

ΔE_{conc} and OCV are summarized in Table S14. The results indicate that OCV is drastically larger than ΔE_{conc}, which means that the contribution of the concentration to the potential shift is negligible.

Table S14. The amount of ferrocyanide/ferricyanide in the anolyte, state of charge, OCP, and the potential shift derived from the concentration difference (ΔE_{conc}) after the thermal regeneration.

cycle	Ferrocyanide / mmol	Ferricyanide / mmol	OCV / mV	ΔE_{conc} / mV
Initial electrolyte	1.5	0		
1st	0.914	0.586	499	22.8
2nd	1.016	0.484	364	38.1
3rd	0.876	0.624	381	17.4
4th	1.079	0.421	368	48.3
5th	0.808	0.692	351	8.00

(1) Wang, H.; Sayed, S. Y.; Luber, E. J.; Olsen, B. C.; Shirurkar, S. M.; Venkatakrishnan, S.; Tefashe, U. M.; Farquhar, A. K.; Smotkin, E. S.; McCreery, R. L.; et al. Redox Flow Batteries: How to Determine Electrochemical Kinetic Parameters. *ACS Nano* **2020**, *14* (3), 2575-2584. DOI: 10.1021/acsnano.0c01281
